# Environmental Contamination and Rapid Evolution of H9N2 Avian Influenza Virus in Live Bird Markets, South Korea, 2023–2025: Implications for Zoonotic Risk

**DOI:** 10.1155/tbed/4393527

**Published:** 2026-11-09

**Authors:** Dong-Yeop Lee, Sori Kim, Sun-Hak Lee, Andrew Y. Cho, Heesu Lee, Nyamsuren Otgontogtokh, Ye-Ram Seo, Kai-Yun Tan, Sae-Hyeon Park, Ji-Yeong Kim, Yongwoo Son, Wonjun Sung, Chang-Seon Song, Dong-Hun Lee

**Affiliations:** ^1^ Wildlife Health Laboratory College of Veterinary Medicine Konkuk University Seoul Republic of Korea konkuk.ac.kr; ^2^ Avian Disease Laboratory College of Veterinary Medicine Konkuk University Seoul Republic of Korea konkuk.ac.kr

**Keywords:** avian influenza, environmental surveillance, genomic analysis, live bird market, phylogenetics

## Abstract

H9N2 avian influenza viruses (AIVs) continue to cause disease in poultry and pose a zoonotic risk. In South Korea, live bird markets (LBMs) serve as evolutionary epicenters for H9N2 AIVs, facilitating interspecies transmission and genetic reassortment. To improve surveillance efficiency, environmental sampling was conducted at two LBMs in Korea between November 2023 and May 2025. A total of 486 samples, comprising 190 poultry swab samples, 179 environmental samples, and 117 tissue samples, were collected. A sample was considered positive when virus isolation in embryonated eggs yielded hemagglutination‐positive allantoic fluid that was subsequently confirmed as AIV by matrix (M)‐gene real‐time reverse transcription PCR (rRT‐PCR). Drinking water showed the highest AIV isolation rate (15.7%). Whole‐genome sequencing of 45 isolates revealed that all viruses belonged to clade B4.6.1 of the B‐lineage (formerly Y280). Phylogenetic analysis suggested the presence of two distinct temporal clusters: the 2023 cluster, which was not detected after November 2023, and the 2024–2025 cluster, which appeared to continue circulating in Korean LBMs. A historical reassortment involving PB1 and PA segments from clade B4.7 H9N2 viruses was inferred to have occurred prior to the introduction of the B‐lineage into Korea. An isolate (N22‐97) exhibited evidence of reassortment between the temporal clusters within clade B4.6.1: its PA segment clustered with the 2024–2025 viruses, whereas the remaining segments clustered with the 2023 viruses. Multiple mammalian‐adaptive mutations (e.g., HA‐226L and PB2‐598I) were identified, with genetic variation observed in drinking water‐derived isolates. Single nucleotide variant (SNV) analysis revealed HA‐A190T/V and HA‐V318I variants, suggesting the presence of mixed viral populations within LBM environments. Sequence‐based antigenic mapping identified five putative antigenic clusters, with most isolates—including the current Korean B‐lineage vaccine strain—grouping within the dominant cluster. These findings highlight drinking water as a useful environmental matrix for AIV monitoring and underscore the value of environmental genomic surveillance for AIV detection in LBMs.

## 1. Introduction

Avian influenza viruses (AIVs) circulate among wild aquatic birds and domestic poultry, where continuous replication and interspecies transmission create opportunities for rapid evolution and zoonotic emergence [1, 2]. Low‐pathogenic AIVs (LPAIVs), particularly H9N2 viruses, have repeatedly demonstrated their importance as reservoirs of internal genes for highly pathogenic AIVs (HPAIVs) and as persistent agents in poultry production systems [3, 4]. H9N2 AIV is the most prevalent LPAIV subtype in poultry, causing significant economic losses across Asia, the Middle East, and Africa [5]. To date, H9N2 viruses have diverged into three major genetic lineages in poultry: the Y‐lineage (formerly referred to as Y439/Korean, h9.3, and American, h9.1–h9.2), the G‐lineage (formerly referred to as G1 or h9.4.1), and the B‐lineage (formerly referred to as BJ/94, Y280, G9, or h9.4.2) [6]. Although classified as LPAIV, H9N2 viruses have also caused sporadic human infections, raising public health concerns due to their zoonotic potential [7]. Since the first documented human H9N2 infection in 1998, a total of 206 human cases, including two fatalities, have been reported as of June 24, 2026 [8]. The high mutation rate and capacity for genetic reassortment of H9N2 viruses enable them to evolve rapidly and contribute internal genes to other AIVs, including H5N1 and H7N9 HPAIVs [9].

In South Korea, H9N2 LPAIVs have circulated endemically in poultry since their first detection in 1996, with the indigenous Y‐lineage predominating until its last documented detection in 2018 [10, 11]. Live bird markets (LBMs) have served as major evolutionary epicenters for H9N2 viruses, with high‐density, multispecies interactions facilitating viral transmission and genetic reassortment among cocirculating viruses [11–16]. Indeed, surveillance conducted from 2006 to 2016 showed that repeated introductions of AIVs from domestic ducks and wild aquatic birds into LBMs generated multiple reassortant genotypes that subsequently became predominant [12]. In 2020, B‐lineage H9N2 viruses were first detected in South Korea and became established in LBMs and poultry holdings, continuing to evolve under local poultry population pressures [12–14]. The Korean B‐lineage H9N2 virus is characterized by frequent bidirectional transmission between LBMs and farms, substantial antigenic divergence from the Y‐lineage, and the presence of mammalian‐adaptive markers, underscoring its ongoing evolution and zoonotic potential [13, 14].

Previous surveillance efforts in LBMs have primarily relied on direct sampling of poultry, which presents several limitations such as sampling bias, high operational costs, and risk of exposure during the handling of infected birds [17, 18]. In contrast, environmental sampling, including water sampling, enables the detection of viruses from shared sources, potentially providing broader coverage of circulating viruses and allowing flock‐level monitoring without the need to handle live birds [19, 20]. Previous studies have demonstrated that drinking water sampling is a practical and noninvasive approach for AIV surveillance in LBMs, frequently yielding comparable or even higher detection and isolation rates than those of bird‐based sampling, thereby highlighting its value as a surveillance strategy [21, 22]. Collectively, these advantages support the use of water‐based environmental sampling as a scalable surveillance tool for detecting AIVs and monitoring their evolutionary dynamics in complex, multihost market systems.

Between November 2023 and May 2025, we conducted environmental monitoring for AIV surveillance in two LBMs located in different regions of South Korea using drinking water and environmental fecal swab samples and compared the results with those obtained from poultry swab and tissue samples. The isolated H9N2 viruses were subjected to next‐generation sequencing (NGS) and subsequently analyzed to elucidate their evolutionary relationships and identify molecular markers associated with mammalian adaptation. This study aimed to assess the utility of environmental surveillance for detecting and monitoring potentially zoonotic AIVs and to provide insights into the genetic and evolutionary dynamics of H9N2 viruses circulating in Korean LBMs.

## 2. Materials and Methods

### 2.1. Sample Collection and Virus Isolation

From November 2023 to May 2025, samples were collected from two LBMs in Korea, hereafter referred to as LBM A in Gyeonggi‐do and LBM B in Chungcheongbuk‐do. Oropharyngeal and cloacal swabs were collected from poultry in the LBMs, including domestic chickens, Pekin ducks, Muscovy ducks, domestic geese, and rock doves, along with environmental samples, including drinking water samples and fecal swabs. At each LBM, sampling was conducted at a single participating vendor, and the same vendor was revisited in every sampling round. During each visit, drinking water and fecal swabs were collected from five different containers and five separate cages, respectively, with containers and cages being selected anew in each round. Because poultry inventory and cage occupancy changed between visits, each round represented a cross‐sectional assessment of the poultry and environmental conditions at that time. Each swab sample was collected using a separate sterile swab and immediately immersed in 1 mL 1 × phosphate‐buffered saline (PBS) after collection, while each drinking water sample was collected in a separate sterile 15 mL conical tube. For poultry carcasses obtained from the LBMs, the trachea and cecal tonsils, selected as target organs for pathogen detection, were excised and pooled for each carcass. Tissue homogenates were prepared by diluting the pooled tissues to 10% (w/v) with 1 × PBS, followed by three freeze–thaw cycles. All samples were stored at –80°C until further analysis. A total of 486 samples were collected by May 19, 2025, comprising 95 oropharyngeal swabs, 95 cloacal swabs, 90 fecal swabs, 89 drinking water samples, and 117 tissue samples.

To minimize cross‐sample contamination, all open‐tube manipulations were performed in a Class II biosafety cabinet. Negative controls were included throughout sample processing, virus isolation, RNA extraction, and real‐time reverse transcription PCR (rRT‐PCR). Each sample was homogenized by vortexing for 1 min and centrifuged at 3000 rpm for 10 min. The supernatant was subsequently collected, filtered through a 0.45‐µm Minisart syringe filter (Sartorius, Göttingen, Germany), and supplemented with gentamicin (final concentration of 1% v/v) before inoculation into the allantoic cavity of 10‐day‐old specific‐pathogen‐free (SPF) embryonated eggs [23]. The inoculated eggs were incubated at 37°C for 72 h, after which the allantoic fluid was harvested and tested for hemagglutination activity. For poultry swab and environmental samples, a second passage was performed to obtain a sufficient virus titer for further analysis. Total RNA was extracted from the hemagglutination‐positive allantoic fluid using the RNeasy Mini Kit (QIAGEN, Hilden, Germany), followed by rRT‐PCR targeting the influenza A matrix (M) gene [24]. All rRT‐PCR‐positive isolates were aliquoted and stored at –80°C for subsequent whole‐genome sequencing.

### 2.2. Whole‐Genome Sequencing and Genome Assembly

For AIV‐positive isolates, all eight gene segments were amplified using the OneTaq One‐Step RT‐PCR Kit (New England Biolabs, Ipswich, MA, USA) with universal primers, as previously described [25]. Successful amplification of all eight gene segments was confirmed by agarose gel electrophoresis. The purified amplicons were prepared for WGS using the Illumina DNA Prep kit and sequenced on the Illumina MiniSeq platform (Illumina Inc., San Diego, CA, USA). The resulting raw reads were quality‐filtered using BBDuk (v38.84) [26] with a Phred score cutoff of Q30 and a minimum read length of 50 bp. Both de novo and reference‐guided genome assemblies were performed. For de novo assembly, contigs were generated from the trimmed reads using Geneious Assembler (v2025.0.3) and identified through BLAST searches against NCBI GenBank to determine reference sequences for each of the eight gene segments. For reference‐guided assembly, the trimmed reads were aligned to the corresponding reference sequences using Minimap2 (v2.24) [27].

### 2.3. Lineage and Clade Classification

The Korean H9N2 isolates were classified using a recently established nomenclature and classification system for A/H9 AIVs [6]. Under this system, the H9 hemagglutinin (HA) gene sequences are classified into three major lineages: Y (formerly Y439), G (formerly G1 or h9.4.1), and B (formerly BJ/94, Y280, or G9). The HA gene sequences obtained in this study were analyzed using the online H9 AIV classification tool (https://nmdc.cn/influvar/tools/H9aiv), which performs multiple sequence alignment and constructs maximum‐likelihood (ML) phylogenetic trees using reference datasets to assign a lineage and clade designation to each query sequence.

### 2.4. Phylogenetic Analysis

For each gene segment, the top 500 BLAST hits for each study sequence were retrieved from the GISAID database (accessed July 6, 2025) [28]. Following alignment with MAFFT (v7.490) [29], identical sequences were removed using ElimDupes (https://www.hiv.lanl.gov/content/sequence/elimdupesv2/elimdupes.html). Preliminary ML trees were inferred using FastTree 2 (v2.1.11) under the GTR + Γ substitution model [30]. Sequences showing poor fit in the root‐to‐tip regression analysis were identified using TempEst (v1.5.3) and excluded from further analysis [31]. Final ML phylogenies were reconstructed using IQ‐TREE 3 (v3.0.1), with ModelFinder used to select the optimal substitution model [32, 33]. Branch support was assessed using 1000 ultrafast bootstrap replicates. The trees were rooted using the best‐fitting root inferred using TreeTime (v0.11.4) [34] and visualized using Interactive Tree of Life (iTOL, v7.2) [35].

The evolutionary history of the HA gene was subsequently reconstructed using Bayesian time‐scaled phylogenetic inference implemented in BEAST (v1.10.4) [36]. Root‐to‐tip regression analysis was performed using TempEst (v1.5.3) to assess the temporal signal [31]. The nucleotide alignment was analyzed using the GTR + Γ substitution model with four discrete gamma rate categories to account for among‐site rate heterogeneity, with an uncorrelated lognormal relaxed molecular clock to accommodate substitution‐rate variation among branches. Demographic history was estimated using a Gaussian Markov Random Field (GMRF) Bayesian skyride coalescent prior [37]. Five independent Markov Chain Monte Carlo (MCMC) runs of 50 million generations were performed, with parameters sampled every 5000 steps. Convergence and effective sample sizes (ESSs) were assessed using Tracer (v1.7.2), with ESS values > 200 considered sufficient [38]. Four MCMC runs that met the convergence and ESS criteria were combined using LogCombiner (v1.10.4) after discarding the first 10% of each run as burn‐in. A maximum clade credibility (MCC) tree was summarized using TreeAnnotator (v1.10.4) with median node heights and visualized using FigTree (v1.4.4).

### 2.5. Reassortment Analysis

A separate dataset for reassortment analysis was constructed by retrieving 23,250 H9N2 virus segment sequences, representing all publicly available sequences in GISAID, collected between January 1, 2019, and July 22, 2025 (accessed July 22, 2025). Sequences were filtered to retain only complete coding sequences (CDSs), and the Korean B‐lineage H9N2 virus sequences obtained in this study were incorporated into the dataset. Multiple sequence alignments were generated for each gene segment using MAFFT (v7.490) [29]. Isolates identified as molecular clock outliers based on the HA segment were excluded from all segment datasets prior to reassortment analysis. Reassortment events were assessed using TreeSort (v0.3.0) [39], with the HA phylogeny designated as the reference tree. The timing of reassortment events was estimated using time‐scaled ML trees generated for each gene segment with TreeTime (v0.11.4) [34].

### 2.6. Amino Acid Analysis

Amino acid substitutions potentially associated with the biological properties of AIVs were initially annotated using FluSurver (https://flusurver.bii.a-star.edu.sg/). HA residue positions were converted to the H3 and H9 numbering schemes using the HA Subtype Numbering Conversion tool provided by the BV‐BRC resource [40]. Substitutions previously reported to have biological significance in H9N2 AIVs were then screened and interpreted based on published studies [41, 42]. Full‐length HA amino acid sequences were analyzed using the NetNGlyc 1.0 server to predict potential N‐linked glycosylation sites [43]. Antigenic sites in the H9 HA protein were annotated based on previous studies [44, 45].

### 2.7. Single Nucleotide Variant (SNV) Analysis

To examine the within‐isolate genetic variation of amino acid substitutions, SNVs were identified from quality‐trimmed (Q30) sequence reads generated after egg amplification using Geneious Prime (v2025.1.3). Reads were mapped to the corresponding consensus sequence of each isolate. Variants were required to have a variant‐calling *p*‐value ≤ 10^−6^ and, when exhibiting > 65% strand bias, a strand‐bias *p*‐value ≥ 10^−5^. Nonsynonymous SNVs with a variant frequency ≥ 3% and a minimum read depth of 200 × were retained using thresholds adapted from previously published criteria [46]. The filtered dataset was subsequently examined for nonsynonymous SNVs affecting amino acid sites previously reported to have biological significance [41, 42].

### 2.8. Sequence‐Based Antigenic Distance Mapping (SBM)

SBM was applied as previously described to evaluate sequence‐inferred antigenic diversity among the HA proteins of Korean B‐lineage H9N2 viruses [47]. HA nucleotide sequences were translated into amino acid sequences and aligned using MAFFT (v7.490) [29]. Antigenic distances (ADs) were calculated based on amino acid mismatches within two H9 antigenic regions: H9‐A (positions 145, 146, 149, 179, 180, 183, 212, 217, and 234; H9 numbering) and H9‐B (positions 72, 115, 120, 135, 139, and 162; H9 numbering) [44, 45]. For each region, the epitope distance (ED) was calculated as the proportion of amino acid mismatches among pairwise‐comparable positions within the epitope, multiplied by 20. The AD between each sequence pair was calculated as the arithmetic mean of the two EDs: AD = (ED_H9‐A + ED_H9‐B)/2. Positions containing gaps or ambiguous residues were excluded. Classical multidimensional scaling (MDS) and goodness‐of‐fit (GOF) metrics were computed in two and three dimensions using the *cmdscale* function with a Cailliez additive correction in R (v4.5.2). Stress was calculated as the normalized root residual sum of squares between the Cailliez‐corrected input distances and the distances fitted in the MDS configuration. Hierarchical clustering was performed on the AD matrix using the Unweighted Pair Group Method with Arithmetic Mean (UPGMA), implemented with the *hclust* function in R. The number of clusters (*k*) was determined by maximizing the average silhouette width over *k* = 2–10, and clusters were assigned using the *cutree* function in R. The resulting dendrogram was exported in Newick format using the *ape* package in R and visualized using FigTree (v1.4.4).

### 2.9. Statistical Analysis

The viral isolation rate for each sample type was calculated as the number of virus isolation‐positive samples divided by the total number of samples tested, and 95% confidence intervals (CIs) were estimated using the Wilson score method. All analyses were performed in R (v4.5.2).

## 3. Results

### 3.1. Virus Detection and Whole‐Genome Sequencing

Overall, 9.3% (45/486; 95% CI, 7.0%–12.2%) of the samples were positive for AIV by virus isolation, followed by M‐gene rRT‐PCR confirmation (Supporting Information 1: Table S1). The AIV isolation rates by sample type were 11.6% (11/95; 95% CI, 6.6%–19.6%) for oropharyngeal swabs, 0% (0/95; 95% CI, 0.0%–3.9%) for cloacal swabs, 7.8% (7/90; 95% CI, 3.8%–15.2%) for fecal swabs, 15.7% (14/89; 95% CI, 9.6%–24.7%) for drinking water samples, and 11.1% (13/117; 95% CI, 6.6%–18.1%) for tissue samples. For LBM A, 11.3% (36/320; 95% CI, 8.2%–15.2%) of the samples were positive, including 19.2% (10/52; 95% CI, 10.8%–31.9%) of oropharyngeal swabs, 0% (0/52; 95% CI, 0.0%–6.9%) of cloacal swabs, 12.0% (6/50; 95% CI, 5.6%–23.8%) of fecal swabs, 14.3% (7/49; 95% CI, 7.1%–26.7%) of drinking water samples, and 11.1% (13/117; 95% CI, 6.6%–18.1%) of tissue samples. All positive tissue samples in this study originated from this site, as carcasses were collected only from LBM A. In LBM B, the overall AIV isolation rate was 5.4% (9/166; 95% CI, 2.9%–10.0%), comprising 2.3% (1/43; 95% CI, 0.4%–12.1%) of oropharyngeal swabs, 0% (0/43; 95% CI, 0.0%–8.2%) of cloacal swabs, 2.5% (1/40; 95% CI, 0.4%–12.9%) of fecal swabs, and 17.5% (7/40; 95% CI, 8.7%–31.9%) of drinking water samples. Complete genomes comprising all eight gene segments were successfully recovered from all 45 isolates by NGS.

### 3.2. Lineage and Clade Classification

Phylogenetic analysis of the HA gene placed all isolates from this study within clade B4.6.1 of the B‐lineage according to the nomenclature system proposed by Fusaro et al. [6] (Figure 1). All Korean B‐lineage H9 HA sequences available in the GISAID database were included in our analysis and were also assigned to clade B4.6.1, suggesting that this clade predominates among the available Korean B‐lineage H9N2 virus sequences (Supporting Information 5: Figure S1).

**Figure 1:**
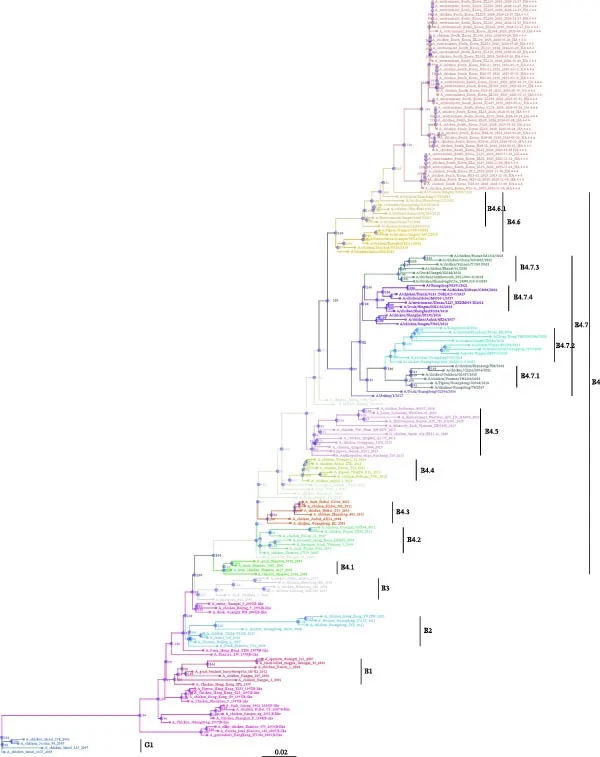
Maximum‐likelihood phylogenetic tree based on the HA gene sequences of A/H9 viruses. The tree was constructed using a B‐lineage‐specific reference dataset according to the classification system proposed by Fusaro et al. Isolates obtained in this study are highlighted in light pink. Bootstrap support values are indicated at the nodes. The scale bar represents the number of nucleotide substitutions per site.

### 3.3. Phylogenetic Analysis

Consistent with the HA lineage classification, the ML phylogenetic tree of the HA gene showed that all H9N2 isolates from this study belonged to the B‐lineage (Figure 2). The isolates formed two distinct temporal clusters corresponding to different sampling periods (2023 and 2024–2025), with a bootstrap support of 97%–99%. Overall, the HA genes of the isolates showed high nucleotide sequence identities (96.79%–100%), with identities of 99.70%–100% among the 2023 isolates and 98.04%–100% among the 2024–2025 isolates. Within individual sampling rounds, isolates obtained from tissue, swab, and environmental samples showed high nucleotide sequence identity (98.51%–100%). The A/chicken/Korea/LBM314/2020 (EPI_ISL_492108) strain, which served as the seed virus for the recombinant inactivated B‐lineage vaccine introduced in early 2023, occupied a basal phylogenetic position relative to the 2023 and 2024–2025 clusters [48]. It showed lower nucleotide identity to the 2024–2025 isolates (97.80%–98.34%) than to the 2023 isolates (98.63%–98.81%). In the HA ML phylogeny, the Korean cluster was most closely related to A/Chicken/Liaoning/20425‐1‐Mix/2020 (EPI_ISL_18116023), which was detected in China in 2020. ML phylogenies of the eight gene segments included sequences from viruses detected in multiple mammalian hosts (including humans, dogs, minks, and others), as well as HPAIVs of multiple subtypes (including H5N1, H5N6, H7N9, and others). Some of these sequences were phylogenetically close to the Korean H9N2 isolates (Supporting Information 6: Figure S2).

**Figure 2:**
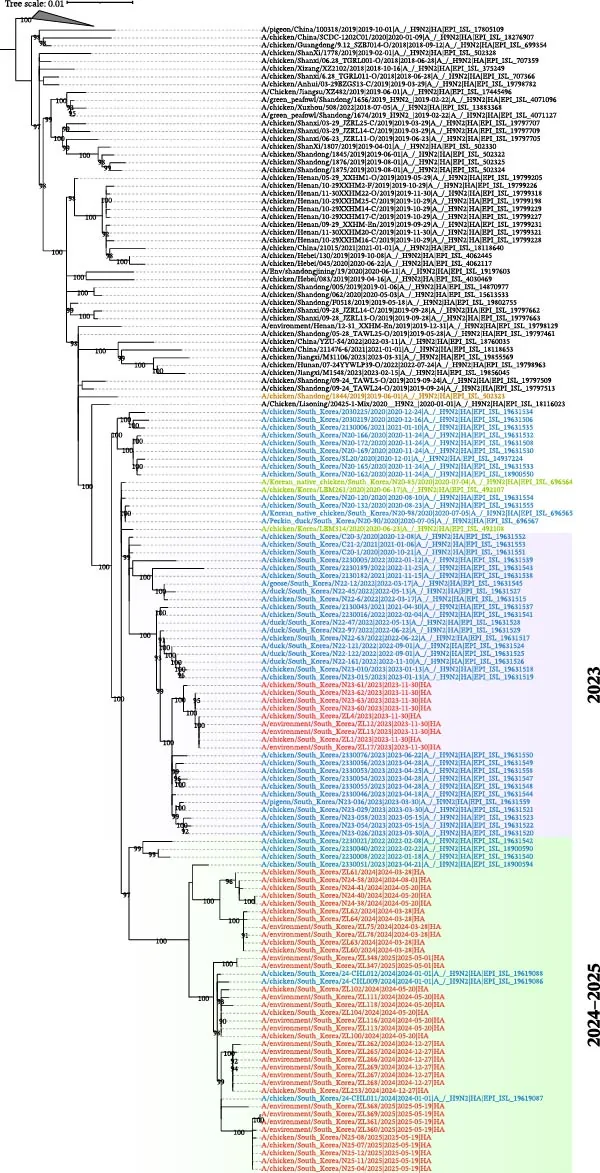
Maximum‐likelihood phylogenetic tree based on the HA gene sequences of B‐lineage H9N2 viruses. Ultrafast bootstrap support values ≥ 90% are indicated at the respective branches. Taxa labels highlighted in red indicate isolates obtained in this study, whereas those highlighted in blue indicate other Korean isolates. The earliest B‐lineage viruses identified in South Korea are highlighted in light green, and the previously proposed origin strain from China (A/chicken/Shandong/1844/2019) is highlighted in ocher. The 2023 and 2024–2025 clusters represent temporal groupings consistent with sampling dates. The tree was rooted using the best‐fitting root inferred by TreeTime.

Bayesian phylogenetic analysis estimated the time to the most recent common ancestor (tMRCA) of all Korean B‐lineage H9N2 viruses included in the analysis to be December 23, 2019 (95% highest posterior density interval [HPDI]: September 21, 2019–March 20, 2020; posterior probability [PP] = 0.995) (Figure 3). The tMRCA of the 2023 cluster was estimated to be August 11, 2020 (95% HPDI: June 5, 2020–October 12, 2020; PP = 0.9936), whereas that of the 2024–2025 cluster was estimated to be December 21, 2020 (95% HPDI: June 23, 2020–May 28, 2021; PP = 0.986). Within the study dataset, the 2023 cluster was not detected after November 2023, whereas the 2024–2025 cluster continued to be detected until the end of the surveillance period in May 2025.

**Figure 3:**
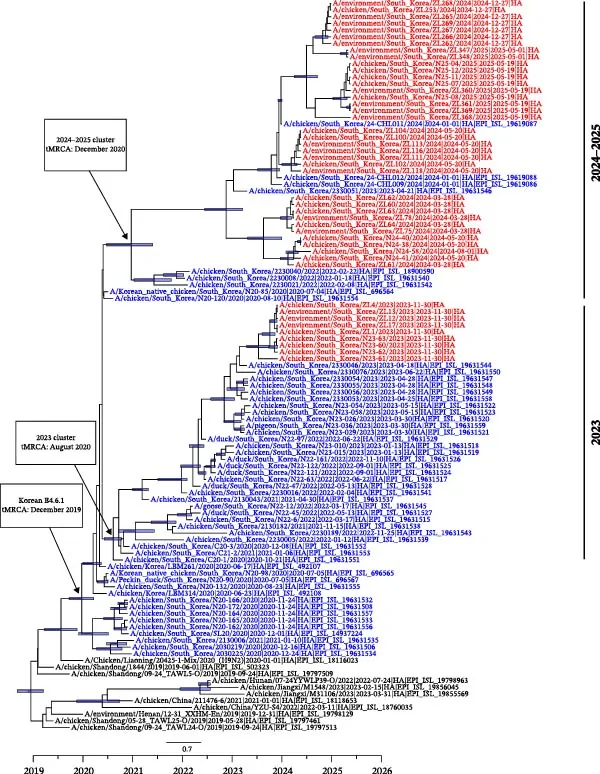
Time‐scaled Bayesian maximum clade credibility tree based on the HA gene sequences of B‐lineage H9N2 viruses. Taxa labels highlighted in red indicate isolates obtained in this study, whereas those highlighted in blue indicate other Korean isolates. Node bars represent the 95% highest posterior density intervals for node ages, with posterior probabilities > 0.5. The 2023 and 2024–2025 clusters represent temporal groupings consistent with sampling dates. The horizontal axis represents calendar time in years.

### 3.4. Reassortment Analysis

Reassortment analysis of 2,341 complete H9N2 genomes using TreeSort identified a distinct reassortment event associated with the Korean B‐lineage H9N2 viruses, involving the PB1 and PA segments between viruses assigned to HA clades B4.6 and B4.7 (Figure 4). Based on the HA phylogeny, this reassortment event was estimated to have occurred in China around April 4, 2017, representing the most recent interclade exchange linked to the Korean B‐lineage H9N2 viruses in the dataset. The PB1 phylogeny showed mixed clustering of viruses assigned to HA clades B4.6 and B4.7 (Supporting Information 7: Figure S3), suggesting that the PB1 segment of the Korean isolates may have been derived from viruses assigned to HA clade B4.7, represented by A/Hubei/1803/2019 (EPI_ISL_499109). The PB1 time‐scaled phylogeny dated the tMRCA of this cluster to September 18, 2017. Similarly, the PA segment of the Korean isolates appeared to have been derived from viruses assigned to HA clade B4.7, represented by A/chicken/China/1104/2019 (EPI_ISL_505807), with the PA tree dating the tMRCA of the corresponding cluster to September 10, 2017. The comparable tMRCA estimates suggest that the PB1 and PA segment exchanges may have occurred contemporaneously in mid‐2017 in China. Extensive gene exchange within clade B4.7 was also observed (Supporting Information 8: Figure S4). Additionally, one previously reported Korean isolate (A/duck/South_Korea/N22‐97/2022) showed reassortment between two temporal clusters within clade B4.6.1, which likely occurred in Korea. Its PA segment phylogenetically grouped with viruses assigned to the 2024–2025 cluster, whereas its remaining seven gene segments grouped with the 2023 cluster (Supporting Information 6: Figure S2).

**Figure 4:**
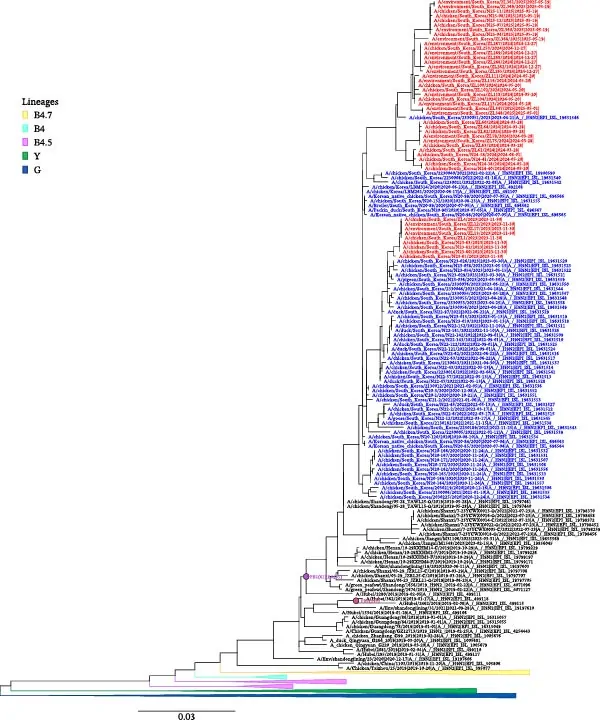
Inferred reassortment events mapped onto the maximum‐likelihood HA phylogeny of clade B4.6 H9N2 viruses collected between January 1, 2019, and July 22, 2025. Clades outside B4.6 that were not closely related to the Korean sequences were collapsed and are represented by colored triangles. Colored circles indicate inferred reassortment events. The corresponding node labels indicate the reassorted gene segment(s) and the number of nucleotide differences from the inferred ancestral sequence. Taxa labels highlighted in red indicate isolates obtained in this study, whereas those highlighted in blue indicate other Korean isolates.

### 3.5. Amino Acid Analysis

All H9N2 AIV isolates in this study were characterized as LPAIVs based on the HA proteolytic cleavage site motif (PSRSSR/GLF). Amino acid analysis identified 17 substitutions previously associated with the biological properties of AIVs across the isolates (Table 1, Supporting Information 2: Table S2). All isolates shared 11 substitutions: HA‐155T, HA‐183N, HA‐212V, HA‐226L, HA‐248D, HA‐306S, HA‐496K (H3 numbering), PB2‐588V, PB1‐368V, PA‐356R, and PA‐409N. Notably, HA‐155T and HA‐190T/V (H3 numbering) were located within the H9‐A antigenic site [44, 45]. LBM314 (the vaccine seed strain) and the 2023 isolates contained PB2‐598V, whereas the 2024–2025 isolates harbored PB2‐598I. The PB2‐598I substitution has been associated with mammalian adaptation [62]. The HA‐190T/V substitution was detected in six isolates: five isolates (N24‐38, N24‐40, N24‐41, ZL111, and ZL118) collected concurrently at LBM A on May 20, 2024, and one (ZL262) at LBM B on December 27, 2024. All isolates analyzed in this study and LBM314 shared an identical predicted HA N‐linked glycosylation profile comprising seven conserved potential sites (Supporting Information 3: Table S3). Six sites were located in the HA1 subunit (positions 21, 73, 128, 289, 296, and 304; H3 numbering), and one was located in the HA2 subunit (position 483; H3 numbering). Compared with the reference strain A/Chicken/Hong Kong/G9/1997 (EPI_ISL_1263), all isolates possessed the HA‐T212V and HA‐P306S substitutions, corresponding to the loss and gain of potential N‐linked glycosylation sites, respectively.

**Table 1:** Molecular markers associated with biological properties identified in Korean B‐lineage H9N2 viruses obtained in this study.

Segment	Substitution	Frequency	Effect	Reference
HA	155T (145)^a^	45/45	Increased virus binding to α2–6	[49, 50]
	183N (173)	45/45	Enhances human‐like receptor binding	[51]
	190T/V (180)	6/45	Enhances binding affinity to mammalian cells and replication in mammalian cells	[52]
	197K (187)	2/45	Increased virus binding to α2–6	[53]
	212V (202)	45/45	Removes a potential N‐glycosylation site	FluSurver^b^
	226L (216)	45/45	Increased virus binding to α2–6, enhanced replication in mammalian cells and ferrets, enhanced contact transmission in ferrets	[54, 55]
	248D (238)	45/45	Increased virus binding to α2–6	[56]
	306S (297)	45/45	Creates a new potential N‐glycosylation site	FluSurver
	318I (309)	2/45	Transmissible among ferrets	[57]
	496K (487)	45/45	Increased virus binding to α2–6	[53]

PB2	9N	1/45	Increased virulence in mice	[58, 59]
	28I	2/45	Decreased polymerase activity in mammalian cell line	[60]
	588V	45/45	Increased polymerase activity and replication in mammalian and avian cell lines, increased virulence in mice	[61]
	598I	36/45	Increased polymerase activity and replication in mammalian cells, increased virulence in mice	[62]

PB1	368V	45/45	Airborne transmissible in ferrets	[63]

PA	356R	45/45	Increased polymerase activity and enhanced replication in mammalian cell line, increased virulence in mice	[64]
	409N	45/45	Characteristic residue of human influenza virus	[65, 66]

Abbreviations: HA, hemagglutinin; PA, polymerase acidic protein; PB1, polymerase basic 1 protein; PB2, polymerase basic 2 protein.

^a^Mature H3 HA numbering (mature H9 HA numbering).

^b^Predicted N‐glycosylation sites using FluSurver.

### 3.6. SNV Analysis

Within‐isolate SNV analysis revealed nonsynonymous variants at several amino acid positions of interest (Table 2). In the HA segment, SNVs resulting in HA‐190T or HA‐190V (H3 numbering) were detected in five isolates, with variant frequencies ranging from 28.8% to 84.6%. Of these, three isolates were collected on May 20, 2024: N24‐41 exhibited the HA‐A190T SNV (variant frequency: 84.6%), whereas ZL104 and ZL113 exhibited the HA‐A190V SNV (variant frequencies: 37.2% and 28.8%, respectively). Other isolates from the same sampling round encoded alanine (A), threonine (T), or valine (V) at this position. The N25‐08 isolate, collected on May 19, 2025, showed the HA‐A190T SNV at a variant frequency of 41.1%, while other isolates collected on the same day retained alanine (A) at this position. The N24‐58 isolate, collected on August 1, 2024, contained nucleotide variants individually corresponding to HA‐190T and HA‐190V (site‐specific variant frequencies: 49.2% and 45.4%, respectively). At HA position 318, the drinking water‐derived isolate ZL369, collected on May 19, 2025, carried the HA‐V318I SNV (variant frequency: 65.4%), whereas the fecal‐derived isolate ZL361, collected from the same sampling round, encoded isoleucine (I); all remaining isolates encoded valine (V).

**Table 2:** Single nucleotide variants and fixed amino acid residues at sites encoding molecular markers associated with biological properties in H9N2 influenza viruses obtained in this study.

Segment^a^	Isolate^b^	Collection date	Sample type	Nt position	Aa position	Nt change	Codon change	Aa change	Variant frequency (%)
HA	N24‐41	May 20, 2024	Tissue	592	190 (180)^c^	G > A	GCG > ACG	A > T	84.6
	ZL104		Oropharyngeal swab	593	190 (180)	C > T	GCG > GTG	A > V	37.2
	ZL113		Fecal swab	593	190 (180)	C > T	GCG > GTG	A > V	28.8
	N24‐38, N24‐40		Tissue	–	190 (180)	–	GTG	V	–^e^
	ZL111		Fecal swab	–	190 (180)	–	ACG	T	–
	ZL118		Drinking water	–	190 (180)	–	GTG	V	–
	The remaining isolates		Oropharyngeal swab, fecal swab, drinking water	–	190 (180)	–	GCG	A	–
	
	N24‐58^d^	Aug 1, 2024	Tissue	592	190 (180)	G > A	GCG > ACG	A > T	49.2
				593	190 (180)	C > T	GCG > GTG	A > V	45.4
	
	N25‐08	May 19, 2025	Tissue	592	190 (180)	G > A	GCG > ACG	A > T	41.1
	The remaining isolates		Tissue, fecal swab, drinking water	–	190 (180)	–	GCG	A	–
	
	ZL369	May 19, 2025	Drinking water	979	318 (309)	G > A	GTT > ATT	V > I	65.4
	ZL361		Fecal swab	–	318 (309)	–	ATT	I	–
	The remaining isolates		Tissue, fecal swab, drinking water	–	318 (309)	–	GTT	V	–

^a^Aa, amino acid; HA, hemagglutinin; Nt, nucleotide.

^b^Isolates were grouped according to sampling date and the presence of SNVs or fixed amino acid substitutions.

^c^Mature H3 HA numbering (mature H9 HA numbering).

^d^N24‐58 was the only isolate recovered on August 1, 2024.

^e^“–” indicates not applicable because no within‐isolate SNV was detected.

### 3.7. SBM

SBM analysis delineated five clusters based on sequence‐derived ADs among Korean B‐lineage H9N2 viruses (Figure 5). The two‐dimensional ordination showed a fair‐to‐good representation of the input distance matrix (stress = 0.1269, GOF = 0.9394). Cluster 1 comprised the majority of strains (71/98, 72.4%). In contrast to the clustering pattern observed in the ML and MCC phylogenetic trees, all 2023 isolates from this study were assigned to Cluster 1, together with the majority of the 2024–2025 isolates. Meanwhile, ZL118 and ZL262 were assigned to Cluster 2, whereas ZL111 and N24‐41 belonged to Cluster 3. Isolates N24‐38 and N24‐40 formed a distinct Cluster 5. The vaccine seed strain A/chicken/Korea/LBM314/2020 was also classified within Cluster 1. All pairwise intercluster AD values were ≥ 1.11, indicating sequence‐derived antigenic separation among Korean B‐lineage H9N2 virus clusters (Supporting Information 4: Table S4). Clusters 4 and 5 showed greater divergence from Clusters 1 and 3, with intercluster AD values ≥ 2.22. When the Y‐lineage reference strain (A/chicken/Korea/01310/2001; EPI_ISL_13845) was included, it was clearly separated from all B‐lineage clusters, with AD values ≥ 11.11, whereas the B‐lineage clusters remained substantially more closely related to one another (Supporting Information 9: Figure S5).

Figure 5Sequence‐based antigenic distance mapping of Korean B‐lineage H9N2 viruses. (A) Hierarchical clustering of HA amino acid sequences based on pairwise antigenic distance calculated from amino acid differences within two defined H9 antigenic regions (H9‐A and H9‐B). The dendrogram was constructed using the unweighted pair group method with arithmetic mean (UPGMA) and partitioned into five predicted antigenic clusters, each indicated by a distinct taxon label color. The vaccine seed strain (A/chicken/Korea/LBM314/2020) is highlighted in red. (B) Two‐dimensional classical multidimensional scaling (MDS) plot of the sequence‐based antigenic distances among the viruses. Each point represents an individual virus sequence, and the proximity between points indicates predicted antigenic similarity. Point colors correspond to the predicted antigenic clusters shown in (A).

**Figure:**
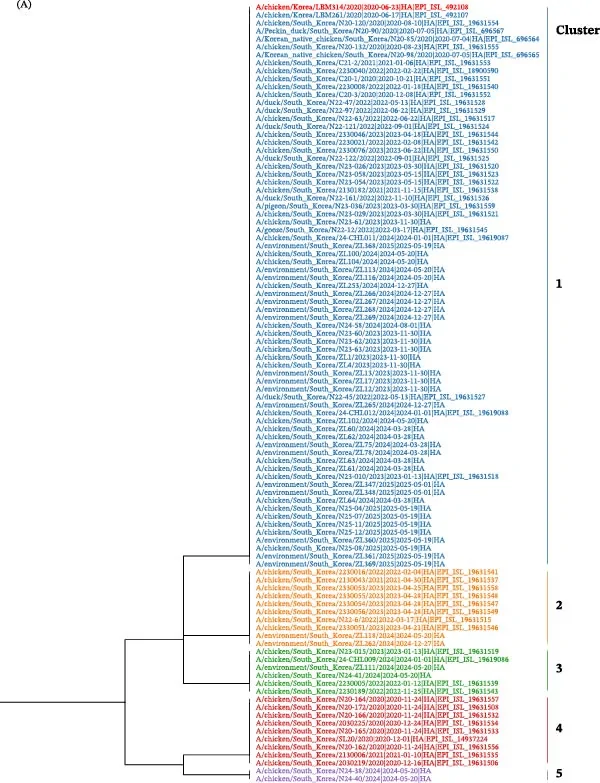

**Figure:**
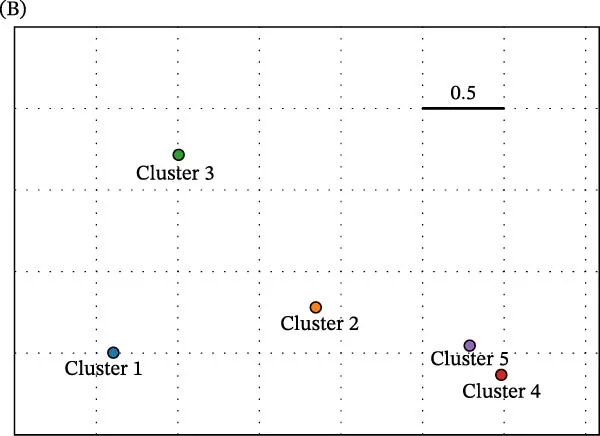

## 4. Discussion

This study provides a comprehensive analysis of H9N2 LPAIVs circulating in Korean LBMs, with a focus on their genetic diversity, transmission dynamics, and mammalian‐adaptive potential. Since its first detection in Korea in June 2020, the B‐lineage H9N2 virus has become established in domestic poultry populations [13, 16, 67]. An important observation of this study was the potential utility of drinking water as a surveillance matrix for AIV detection in LBMs. Drinking water had the highest observed isolation rate among the matrices examined (14/89, 15.7%; 95% CI, 9.6%–24.7%) and yielded AIV isolates in every isolation‐positive sampling round except for the round in which only the tissue‐derived N24‐58 was isolated. On May 1, 2025, drinking water was the only matrix from which AIV was successfully isolated (ZL347 and ZL348). Previous studies have similarly identified drinking water as one of the most sensitive matrices for AIV surveillance in high‐density poultry environments [21, 22, 68–70]. Viruses were also frequently recovered from oropharyngeal swabs but not from cloacal swabs. This pattern is consistent with the preferential replication of B‐lineage H9N2 viruses in the respiratory tract rather than the intestinal tract compared to viruses of the Y‐lineage [11]. Furthermore, H9N2 viruses recovered from tissue, swab, and environmental samples during the same sampling rounds were genetically highly similar, sharing 98.51%–100% HA nucleotide sequence identity. These findings suggest that viruses isolated from drinking water may reflect those circulating concurrently among birds in the market. Although environmental surveillance in wild bird habitats often requires additional virus‐concentration procedures [71], AIVs were successfully recovered from drinking water in this study through two egg passages without prior concentration. This may have been facilitated by the high density of poultry in LBMs and the continuous exposure of shared drinking water to virus‐shedding birds, potentially promoting virus accumulation and persistence. Supporting this, an experimental study demonstrated that the H9N2 virus can remain viable in poultry drinking water for up to 48 h, particularly in the presence of organic debris [21].

All Korean B‐lineage H9N2 isolates analyzed in this study fall within clade B4.6.1 but are phylogenetically distinct from the contemporary Chinese strains included in our analysis. Together with previous reports suggesting a single introduction from China and frequent viral exchange between LBMs and poultry farms, these findings are consistent with the introduction of an ancestral clade B4.6 virus from China, followed by endemic evolution and diversification within Korea [11, 13, 14]. The HA phylogeny identified two temporally distinct groups: the 2023 and 2024–2025 clusters. Viruses belonging to the 2024–2025 cluster were detected until the end of the surveillance period in May 2025, whereas the 2023 cluster was not detected after November 2023. In addition, genetically related viruses were detected in two LBMs approximately 100 km apart, suggesting that poultry trade and distribution networks may contribute to the long‐distance dissemination of specific H9N2 lineages [72]. Bayesian time‐scaled phylogenetic analysis estimated the tMRCAs of the 2023 and 2024–2025 clusters to be in August and December 2020, respectively. These estimates suggest that the two temporal clusters emerged during the early circulation of B‐lineage H9N2 viruses in Korea [13]. The relatively long branches leading to the 2024–2025 isolates, together with the estimated tMRCA, suggest prolonged unsampled circulation or the existence of uncharacterized intermediate viruses before detection. Collectively, these findings indicate that multiple genetically divergent groups within clade B4.6.1 have circulated in Korean LBMs, highlighting the continued evolution and persistence of H9N2 viruses in these environments.

Phylogenetic incongruence and TreeSort analysis provided evidence for reassortment among Korean B‐lineage H9N2 viruses. The differing positions of the 2022 isolate N22‐97 across segment‐specific phylogenetic trees were consistent with reassortment between viruses belonging to the two temporal clusters within the Korean clade B4.6.1. TreeSort analysis also inferred a historical interclade reassortment involving the PB1 and PA segments in the ancestry of Korean B‐lineage H9N2 viruses. This event was estimated to have occurred around 2017 and may have involved viruses assigned to HA clade B4.7 or a related lineage in China. However, these inferences should be interpreted cautiously because they depend on the currently available sequence dataset and may be refined as additional data become available. H9N2 viruses are recognized as major donors of internal gene segments to zoonotic AIVs, including H5N1, H5N6, H7N9, and H10N8 viruses [73–79]. A previous study also identified H9N2‐derived internal genes in human‐infecting H5N1 and H5N8 viruses, including particularly high‐risk strains from Guangdong Province, South China [77], which are phylogenetically linked to the clades B4.6 and B4.7. Collectively, these findings underscore the importance of continued surveillance for reassortment at both the intraclade and interclade levels among Korean B‐lineage H9N2 viruses.

Although no human H9N2 infection has been reported in Korea, zoonotic infections continue to occur globally, primarily in China [80–82]. Phylogenetic analyses have shown that most human‐origin H9N2 viruses belong to the Chinese clade B4.7, with a smaller proportion belonging to clade B4.6 [6]. The Korean H9N2 viruses analyzed in this study did not cluster with viruses recovered from human infection cases included in our analysis; nevertheless, several amino acid substitutions previously associated with mammalian adaptation were identified in our isolates. A previous study reported that substitutions such as HA‐H183N, HA‐E190T, HA‐T192R, HA‐Q226L (H3 numbering), NA‐Δ62‐64, PB2‐A588V, PB2‐K702R, PB1‐I368V, PA‐K356R, PA‐S409N, and M1‐R95K occurred more frequently in human‐origin H9N2 viruses than in avian‐origin viruses [83]. Several of these substitutions, including HA‐183N, HA‐190T, HA‐226L, PB2‐588V, PB1‐368V, PA‐356R, and PA‐409N, were also identified in our isolates. Eleven substitutions were conserved across all sequences analyzed, including HA‐183N, HA‐226L, PB2‐588V, PB1‐368V, PA‐356R, and PA‐409N, all of which have previously been reported to increase in prevalence over time [41]. HA‐183N and HA‐226L are of particular interest because previous studies have associated these residues with increased binding affinity for α2,6‐linked sialic acid receptors [16, 41], which are predominantly expressed in the human upper respiratory tract [84]. Given that receptor‐binding affinity is an important factor in viral adaptation to humans [85], the presence of these substitutions in both our isolates and viruses recovered from human H9N2 cases [7, 83] warrants continued monitoring of their potential zoonotic relevance. The PB2‐V598I substitution distinguished the two temporal clusters: valine (V) was present in the 2023 isolates and the vaccine strain (LBM314), whereas isoleucine (I) was present in the 2024–2025 isolates. PB2‐598I has previously been associated with enhanced polymerase activity, increased replication in mammalian cells, and greater virulence in mice [62]. Its consistent presence in the 2024–2025 isolates may therefore represent a cluster‐associated molecular change with potential relevance to mammalian adaptation, although its phenotypic effect in Korean H9N2 viruses remains to be determined. Five isolates (N24‐38, N24‐40, N24‐41, ZL111, and ZL118) collected from the same site on May 20, 2024, harbored HA‐190T/V substitutions, which have been associated with increased receptor binding and replication in mammalian cells [52]. The detection of alanine (A), threonine (T), and valine (V) at HA position 190 across isolates collected from the same site indicates genetic heterogeneity among cocirculating H9N2 viruses.

SNV analysis identified within‐isolate variation at HA position 190 in five isolates collected from multiple sample types and sampling dates. Variants at this position were detected intermittently throughout the sampling period, suggesting recurrent emergence of this polymorphism within the LBM system. Notably, three isolates collected in May 2024 (N24‐41, ZL104, and ZL113) exhibited within‐isolate variation at this position, whereas contemporaneous isolates showed no detectable within‐isolate variation and appeared to be fixed for alanine (A), valine (V), or threonine (T). In addition, the fecal‐derived isolate ZL361 carried the HA‐V318I substitution, whereas the contemporaneous drinking water‐derived isolate ZL369 contained a mixed V/I population at the same position, with isoleucine (I) accounting for 65.4% of the sequence reads. These observations suggest that genetically heterogeneous viral populations may circulate within LBM environments and that environmental matrices, including feces and drinking water, may contain viral populations originating from multiple birds. Such viral diversity could contribute to persistence, adaptation, and genetic exchange within complex LBM transmission networks. Similar patterns of mixed viral populations and interspecies viral exchange have been reported in LBMs across Asia, supporting their role as ecological hubs for AIV evolution and reassortment [5, 86, 87]. Repeated mixing of viruses in dense, multispecies market environments may therefore create opportunities for the emergence of novel or locally adapted variants, some of which could have zoonotic implications. Nevertheless, these SNV patterns should be interpreted cautiously because they were identified after egg amplification, which may have altered the presence or relative abundance of variants originally present in the samples.

Since early 2023, a recombinant inactivated vaccine against B‐lineage H9N2 AIVs has been used in Korea, with A/chicken/Korea/LBM314/2020 serving as the vaccine seed strain [48]. SBM analysis assigned most Korean B‐lineage H9N2 viruses analyzed (71/98, 72.4%), including the vaccine strain LBM314, to the predominant predicted antigenic cluster (Cluster 1). This finding suggests that the vaccine strain remains antigenically related to a substantial proportion of the sampled viruses based on sequence‐derived predictions, consistent with previous observations [14]. Nevertheless, phylogenetic analyses indicated increasing genetic divergence between the vaccine strain and viruses collected in 2024–2025, reflecting the continued evolution of Korean B‐lineage H9N2 viruses. This divergence, together with the sporadic detection of other predicted antigenic clusters with intercluster AD values >2, suggests ongoing genetic and potential antigenic diversification. Such changes may gradually reduce vaccine‐induced protection over time [88], underscoring the need for periodic re‐evaluation of the protective efficacy of the current vaccine and timely consideration of updates to the vaccine seed strain. However, because the antigenic relationships identified in this study were inferred computationally rather than experimentally validated, they should be regarded as predictions requiring further confirmation. Accordingly, the continued integration of genomic and antigenic surveillance into vaccination policy will be important for detecting potential antigenic drift and supporting evidence‐based vaccine strain selection.

Environmental sampling offers several advantages, including early detection, broad population coverage, and the ability to detect viral circulation among asymptomatic carriers [19, 89]. As a shared medium accessed by multiple hosts, drinking water can facilitate the accumulation of distinct viral populations, thereby serving as an integrated indicator of viral activity at the market level [20, 21, 90]. However, the representativeness of environmental samples may vary according to viral shedding patterns, including differences between oropharyngeal and cloacal shedding. Moreover, individual‐level information on the species, age, and health status of the source bird is generally unavailable [19, 21]. Nevertheless, environmental monitoring remains a valuable component of AIV surveillance in LBMs, potentially enabling viral circulation to be identified before overt clinical outbreaks occur. Following confirmed detection in environmental samples, targeted sampling of individual birds and the prompt implementation of appropriate biosecurity measures should be considered to limit further viral transmission within and beyond the LBM system.

## 5. Conclusion

Our findings indicate that integrating environmental sampling with whole‐genome sequencing can strengthen AIV surveillance in LBMs by facilitating the timely detection and genomic characterization of circulating viruses and providing insights into viral evolution and potential host adaptation in high‐density environments. The high isolation rate from drinking water, together with the high sequence similarity between swab‐ and drinking water‐derived isolates, supports its utility as a practical complementary matrix for AIV surveillance. Given the close interactions between humans and poultry within the shared environments of LBMs, the detection of H9N2 viruses carrying molecular markers associated with mammalian adaptation warrants continued attention from a One Health perspective. Broader genomic surveillance across geographic regions and host species, complemented by phenotypic and antigenic characterization, will be needed to further clarify the evolutionary dynamics of H9N2 viruses and inform evidence‐based risk assessment and mitigation for both animal and human health.

## Funding

This research was financially supported by Konkuk University in 2025.

## Conflicts of Interest

The authors declare no conflicts of interest.

## Supporting Information

Additional supporting information can be found online in the Supporting Information section.

## Supporting information


**Supporting Information 1** Table S1. Korean B‐lineage H9N2 viruses isolated and sequenced in this study.

**Supporting Information 2** Table S2. Molecular markers associated with biological properties identified in Korean B‐lineage H9N2 viruses obtained in this study, listed for each isolate.

**Supporting Information 3** Table S3. Predicted N‐linked glycosylation profiles of Korean B‐lineage H9N2 viruses analyzed in this study.

**Supporting Information 4** Table S4. Pairwise sequence‐based antigenic distances (ADs) among the five predicted antigenic clusters of Korean B‐lineage H9N2 viruses and the Y‐lineage reference strain.

**Supporting Information 5** Figure S1. Maximum‐likelihood phylogenetic tree based on the HA gene sequences of A/H9 viruses, including all Korean B‐lineage H9N2 viruses available in GISAID (accessed July 6, 2025). The tree was constructed using a B‐lineage‐specific reference dataset according to the classification system proposed by Fusaro et al. B‐lineage isolates from South Korea are highlighted in ocher. Bootstrap support values are indicated at the nodes. The scale bar represents the number of nucleotide substitutions per site.

**Supporting Information 6** Figure S2. Maximum‐likelihood phylogenetic trees based on the eight gene segments of B‐lineage H9N2 viruses. Ultrafast bootstrap support values ≥ 90% are indicated at the respective branches. Taxa labels highlighted in red indicate isolates obtained in this study, whereas those highlighted in blue indicate other Korean isolates. The earliest B‐lineage viruses identified in South Korea are highlighted in light green, and the previously proposed origin strain from China (A/chicken/Shandong/1844/2019) is highlighted in ocher. HPAIVs are marked with triangles, and viruses isolated from mammalian hosts (including humans) are marked with squares. Each tree was rooted using the best‐fitting root inferred by TreeTime.

**Supporting Information 7** Figure S3. Maximum‐likelihood phylogenetic trees based on the PB1 (A) and PA (B) gene segments of H9N2 viruses collected between January 1, 2019, and July 22, 2025. Viruses are color‐coded according to their HA lineage and clade assignments; "Korean" denotes the Korean B‐lineage clade B4.6.1 viruses. Clades that are not closely related to the Korean sequences were collapsed and represented by triangles.

**Supporting Information 8** Figure S4. Reassortment events inferred using TreeSort and mapped onto the maximum‐likelihood phylogenetic tree based on the HA sequences of H9N2 viruses collected between January 1, 2019, and July 22, 2025. Viruses are color‐coded according to their HA lineage and clade assignments. Colored circles indicate inferred reassortment events.

**Supporting Information 9** Figure S5. Three‐dimensional classical multidimensional scaling (MDS) plot of sequence‐based antigenic distances among Korean B‐lineage H9N2 viruses and the Y‐lineage reference strain (A/chicken/Korea/01310/2001). Each point represents an individual virus sequence, and the proximity between points indicates predicted antigenic similarity; Clusters 1–5 correspond to the five predicted antigenic clusters of the B‐lineage viruses, and Cluster 6 corresponds to the Y‐lineage reference strain.

## Data Availability

The data that support the findings of this study are openly available in NCBI GenBank at https://www.ncbi.nlm.nih.gov/genbank/, Accession Numbers PV694997‐PV695188, PV717818‐PV717833, and PX129646‐PX129797. The same sequences are also available in the GISAID EpiFlu database at https://gisaid.org/, Accession Numbers EPI_ISL_20134379, EPI_ISL_20172701‐20172705, EPI_ISL_20172711‐20172717, EPI_ISL_20243514‐20243515, and EPI_ISL_20252418‐20252447.
