# Supplementary material for: Environmental Contamination and Rapid Evolution of H9N2 Avian Influenza Virus in Live Bird Markets, South Korea, 2023–2025: Implications for Zoonotic Risk
Source: Transbound Emerg Dis. 2026 Nov 9;2026:4393527. doi: 10.1155/tbed/4393527 (PMC13748803; doi:10.1155/tbed/4393527)
Supplement: Supplementary file 1 [file TBED-2026-4393527-s002.pdf]

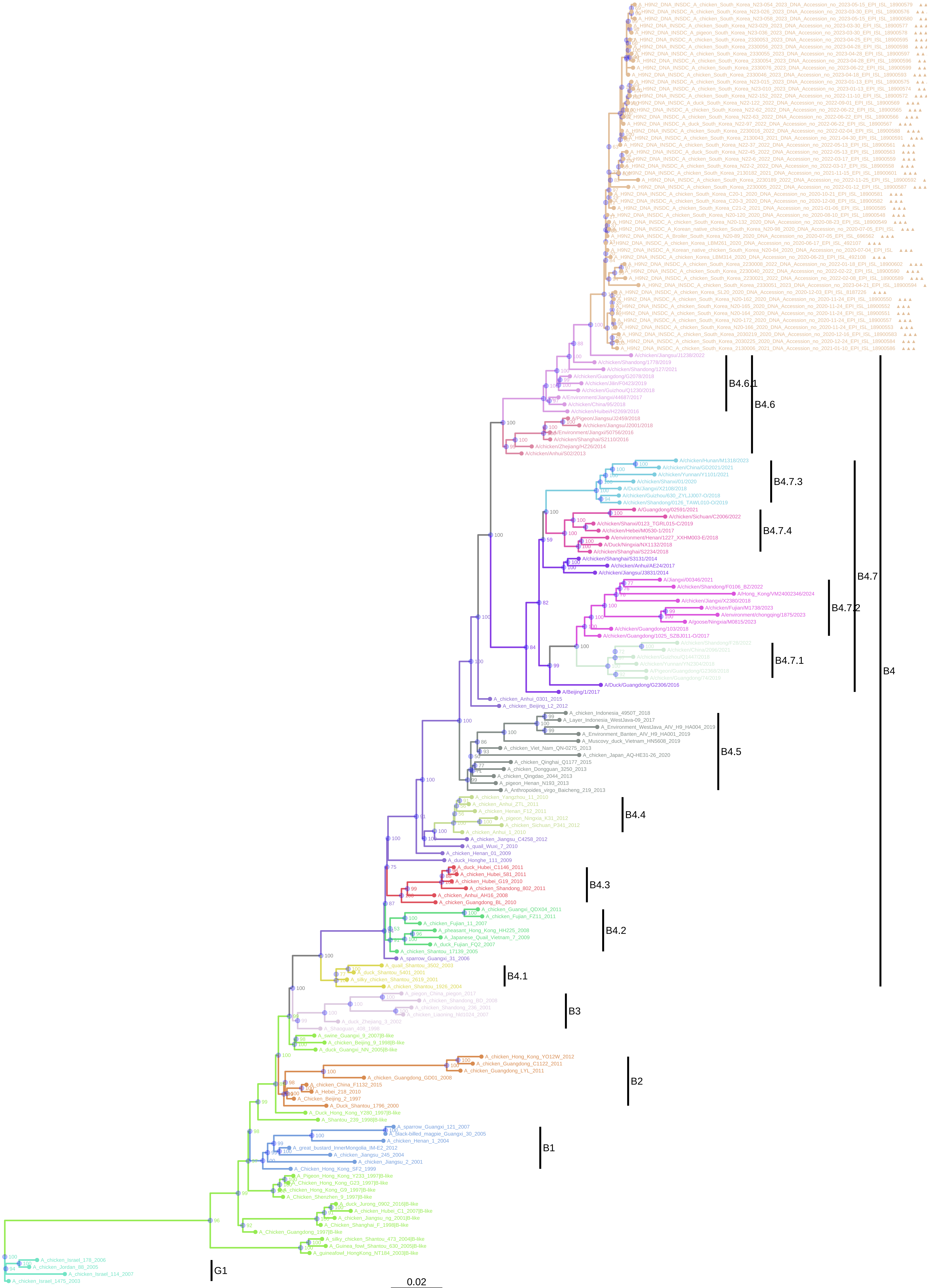

A/chicken/Jiangsu/J1238/2022
A/chicken/Shandong/1778/2019
A/chicken/Shandong/127/2021
A/chicken/Guangdong/G2078/2018
A/chicken/Jilin/F0423/2019
A/chicken/Guizhou/Q1230/2018
A/Environment/Jiangxi/44687/2017
A/chicken/China/95/2018
A/chicken/Hubei/H2269/2016
A/Pigeon/Jiangsu/J2459/2018
A/chicken/Jiangsu/J2001/2018
A/Environment/Jiangxi/50756/2016
A/chicken/Shanghai/S2110/2016
A/chicken/Zhejiang/HZ26/2014
A/chicken/Anhui/S02/2013
A/chicken/Hunan/M1318/2023
A/chicken/China/GD2021/2021
A/chicken/Yunnan/Y1101/2021
A/chicken/Shanxi/01/2020
A/Duck/Jiangxi/X2108/2018
A/chicken/Guizhou/630_ZYLJJ007-O/2018
A/chicken/Shandong/0126_TAWL010-O/2019
A/Guangdong/02591/2021
A/chicken/Sichuan/C2006/2022
A/chicken/Shanxi/0123_TGRL015-C/2019
A/chicken/Hebei/M0530-1/2017
A/environment/Henan/1227_XXHM003-E/2018
A/Duck/Ningxia/NX1132/2018
A/chicken/Shanghai/S2234/2018
A/chicken/Shanghai/S3131/2014
A/chicken/Anhui/AE24/2017
A/chicken/Jiangsu/J3831/2014
A/Jiangxi/00346/2021
A/chicken/Shandong/F0106_BZ/2022
A/Hong_Kong/VM24002346/2024
A/chicken/Jiangxi/X2380/2018
A/chicken/Fujian/M1739/2023
A/environment/chongqing/1875/2023
A/goose/Ningxia/M0815/2023
A/chicken/Guangdong/103/2018
A/chicken/Guangdong/1025_SZBJ011-O/2017
A/chicken/Shandong/F28/2022
A/chicken/China/2096/2021
A/chicken/Guizhou/Q1447/2018
A/chicken/Yunnan/YN2304/2018
A/Pigeon/Guangdong/G2368/2018
A/chicken/Guangdong/74/2019
A/Duck/Guangdong/G2306/2016
A/Beijing/1/2017
A_chicken_Anhui_0301_2015
A_chicken_Beijing_L2_2012
A_chicken_Indonesia_4950T_2018
A_Layer_Indonesia_WestJava-09_2017
A_Environment_WestJava_AIV_H9_HA004_2019
A_Environment_Banten_AIV_H9_HA001_2019
A_Muscovy_duck_Vietnam_HN5608_2019
A_chicken_Viet_Nam_QN-0275_2013
A_chicken_Japan_AQ-HE31-26_2020
A_chicken_Qinghai_Q1177_2015
A_chicken_Dongguan_3250_2013
A_chicken_Qingdao_2044_2013
A_pigeon_Henan_N193_2013
A_Anthropoides_virgo_Baicheng_219_2013
A_chicken_Yangzhou_11_2010
A_chicken_Anhui_ZTL_2011
A_chicken_Henan_F12_2011
A_pigeon_Ningxia_K31_2012
A_chicken_Sichuan_P341_2012
A_chicken_Anhui_1_2010
A_chicken_Jiangsu_C4258_2012
A_quail_Wuxi_7_2010
A_chicken_Henan_01_2009
A_duck_Honghe_111_2009
A_duck_Hubei_C1146_2011
A_chicken_Hubei_581_2011
A_chicken_Hubei_G19_2010
A_chicken_Shandong_802_2011
A_chicken_Anhui_AH16_2008
A_chicken_Guangdong_BL_2010
A_chicken_Guangxi_QDX04_2011
A_chicken_Fujian_FZ11_2011
A_chicken_Fujian_11_2007
A_pheasant_Hong_Kong_HH225_2008
A_Japanese_Quail_Vietnam_7_2009
A_duck_Fujian_FQ2_2007
A_chicken_Shantou_17139_2005
A_sparrow_Guangxi_31_2006
A_quail_Shantou_3502_2003
A_duck_Shantou_5401_2001
A_silky_chicken_Shantou_2619_2001
A_chicken_Shantou_1926_2004
A_pegon_China_pigeon_2017
A_chicken_Shandong_BD_2008
A_chicken_Shandong_236_2001
A_chicken_Liaoning_hld1024_2007
A_duck_Zhejiang_3_2002
A_Shaoguan_408_1998
A_swine_Guangxi_9_2007|B-like
A_chicken_Beijing_9_1998|B-like
A_duck_Guangxi_NN_2005|B-like
A_chicken_Hong_Kong_YO12W_2012
A_chicken_Guangdong_C1122_2011
A_chicken_Guangdong_LYL_2011
A_chicken_Guangdong_GD01_2008
A_chicken_China_F1132_2015
A_Hebei_218_2010
A_Chicken_Beijing_2_1997
A_Duck_Shantou_1796_2000
A_Duck_Hong_Kong_Y280_1997|B-like
A_Shantou_239_1998|B-like
A_sparrow_Guangxi_121_2007
A_black-billed_magpie_Guangxi_30_2005
A_chicken_Henan_1_2004
A_great_bustard_InnerMongolia_IM-E2_2012
A_chicken_Jiangsu_245_2004
A_chicken_Jiangsu_2_2001
A_Chicken_Hong_Kong_SF2_1999
A_Pigeon_Hong_Kong_Y233_1997|B-like
A_Chicken_Hong_Kong_G23_1997|B-like
A_chicken_Hong_Kong_G9_1997|B-like
A_Chicken_Shenzhen_9_1997|B-like
A_duck_Jurong_0902_2016|B-like
A_chicken_Hubei_C1_2007|B-like
A_chicken_Jiangsu_ng_2001|B-like
A_Chicken_Shanghai_F_1998|B-like
A_Chicken_Guangdong_1997|B-like
A_silky_chicken_Shantou_473_2004|B-like
A_Guinea_fowl_Shantou_630_2005|B-like
A_guineafowl_HongKong_NT184_2003|B-like
A_chicken_Israel_178_2006
A_chicken_Jordan_88_2005
A_chicken_Israel_114_2007
A_chicken_Israel_1475_2003
B4.6.1
B4.6
B4.7.3
B4.7.4
B4.7
B4.7.2
B4.7.1
B4
B4.5
B4.4
B4.3
B4.2
B4.1
B3
B2
B1
G1
0.02
