# Supplementary material for: Environmental Contamination and Rapid Evolution of H9N2 Avian Influenza Virus in Live Bird Markets, South Korea, 2023–2025: Implications for Zoonotic Risk
Source: Transbound Emerg Dis. 2026 Nov 9;2026:4393527. doi: 10.1155/tbed/4393527 (PMC13748803; doi:10.1155/tbed/4393527)
Supplement: Supplementary file 2 [file TBED-2026-4393527-s003.pdf]

HA

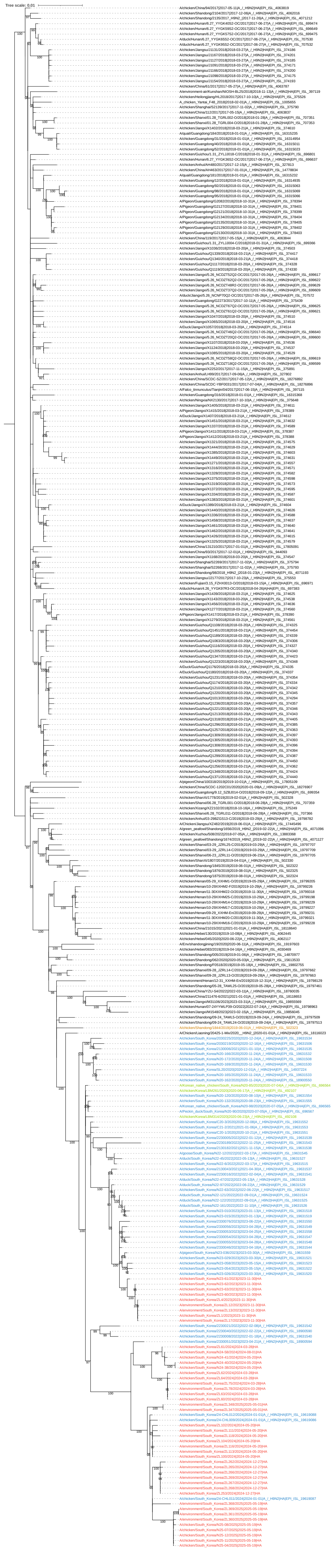

NA

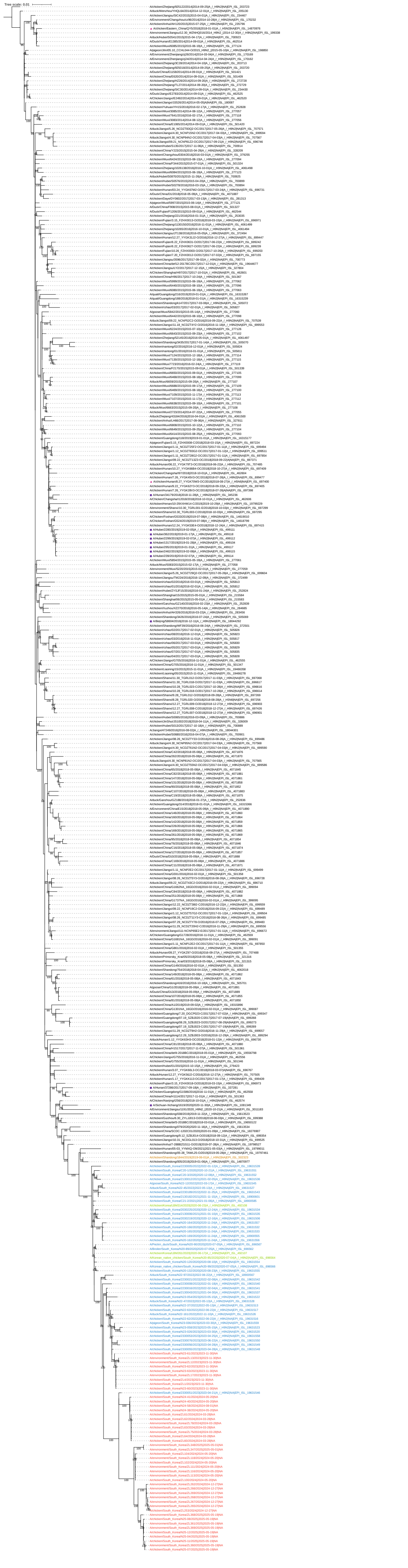

PB2

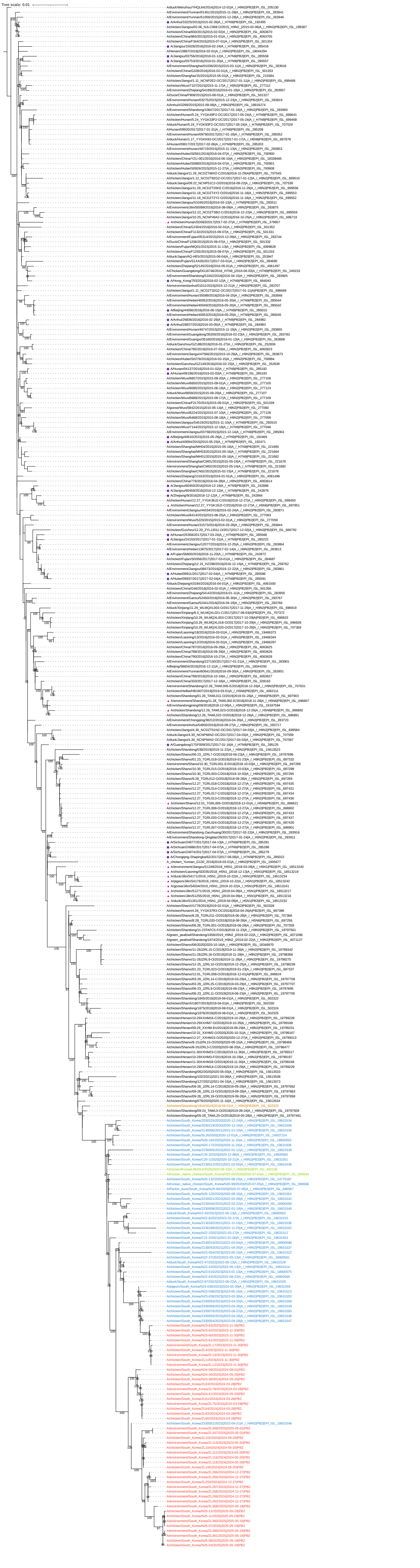

PB1

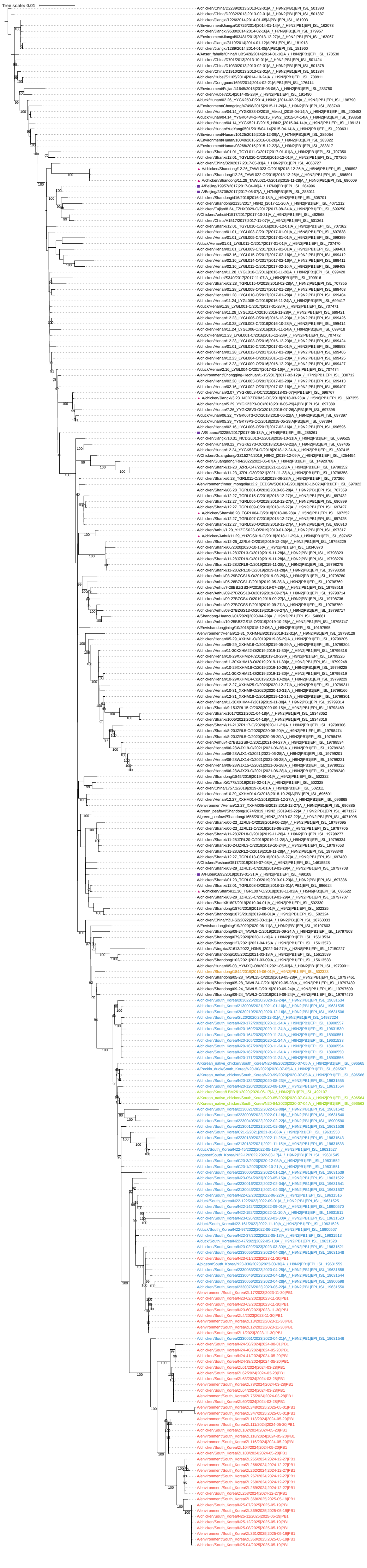

PA

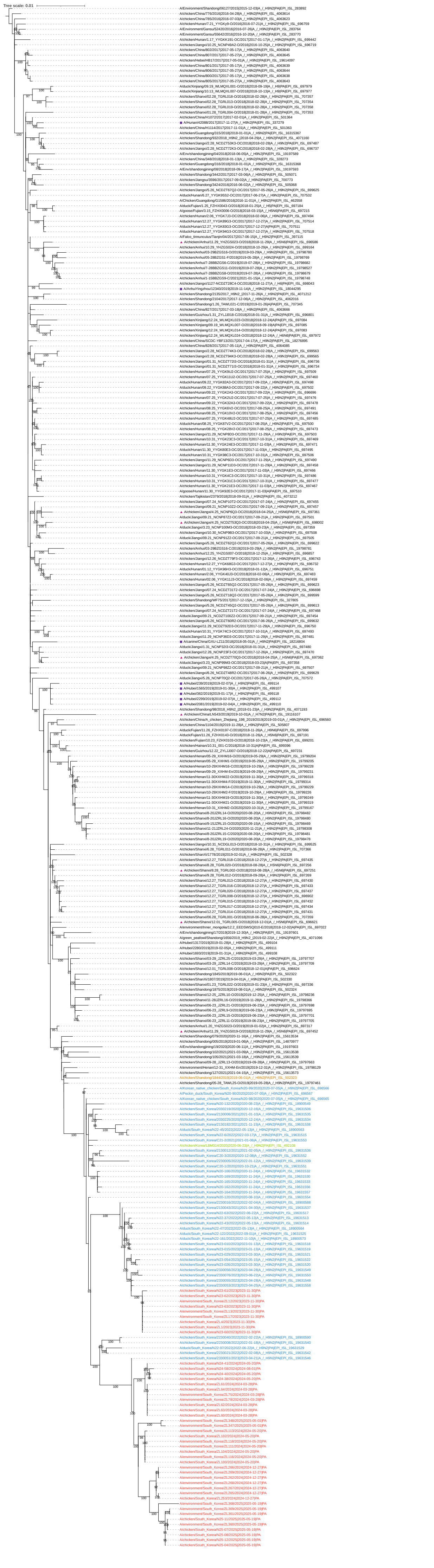

NP

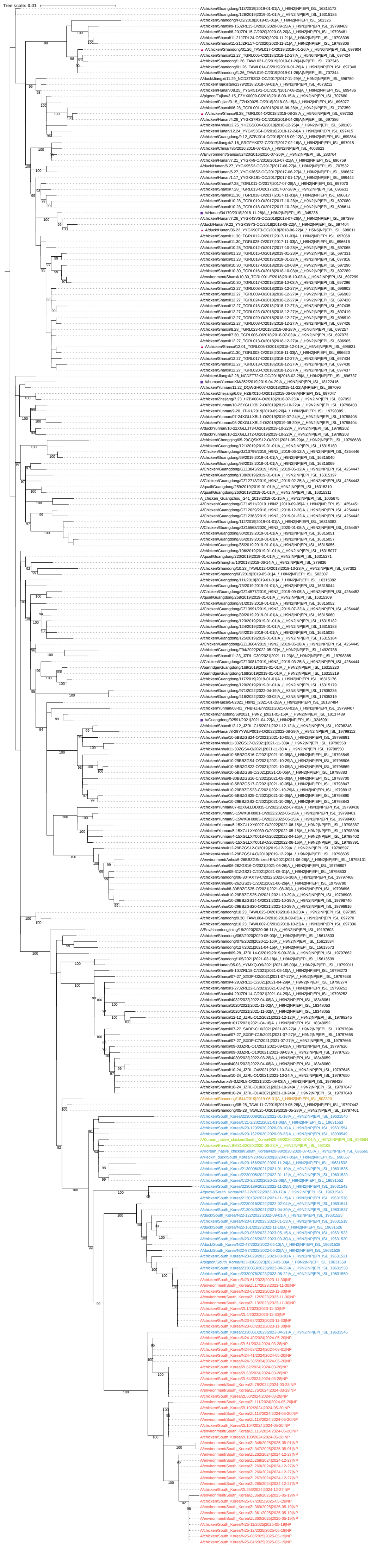

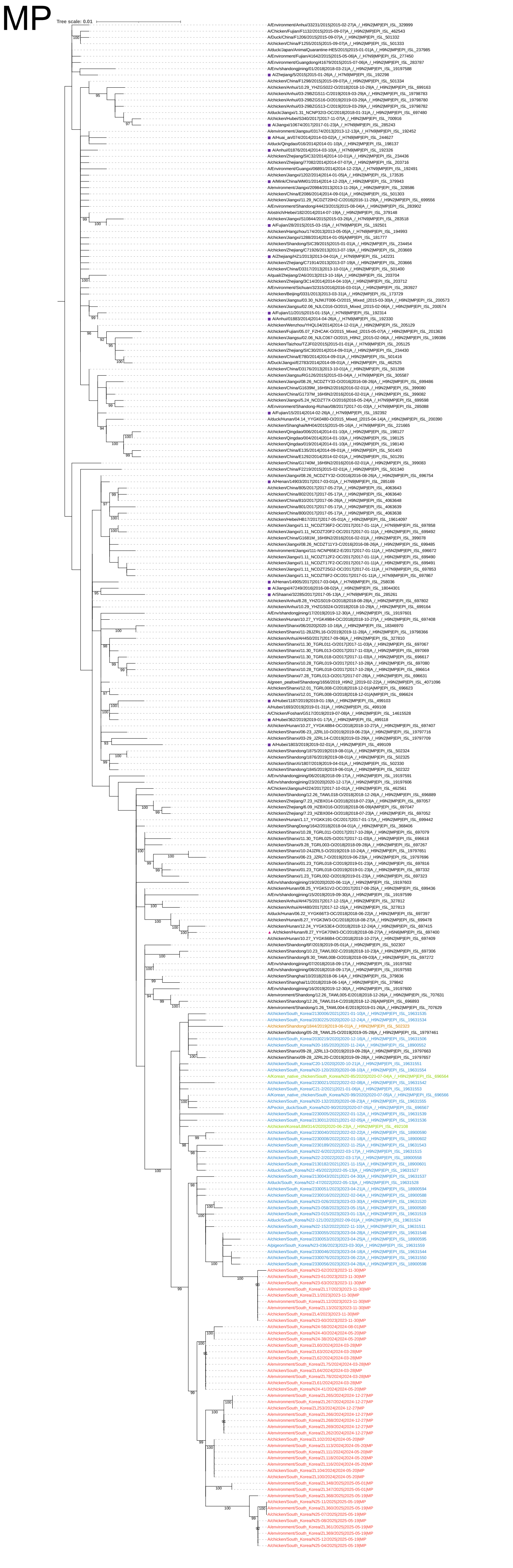
MP
Tree scale: 0.01

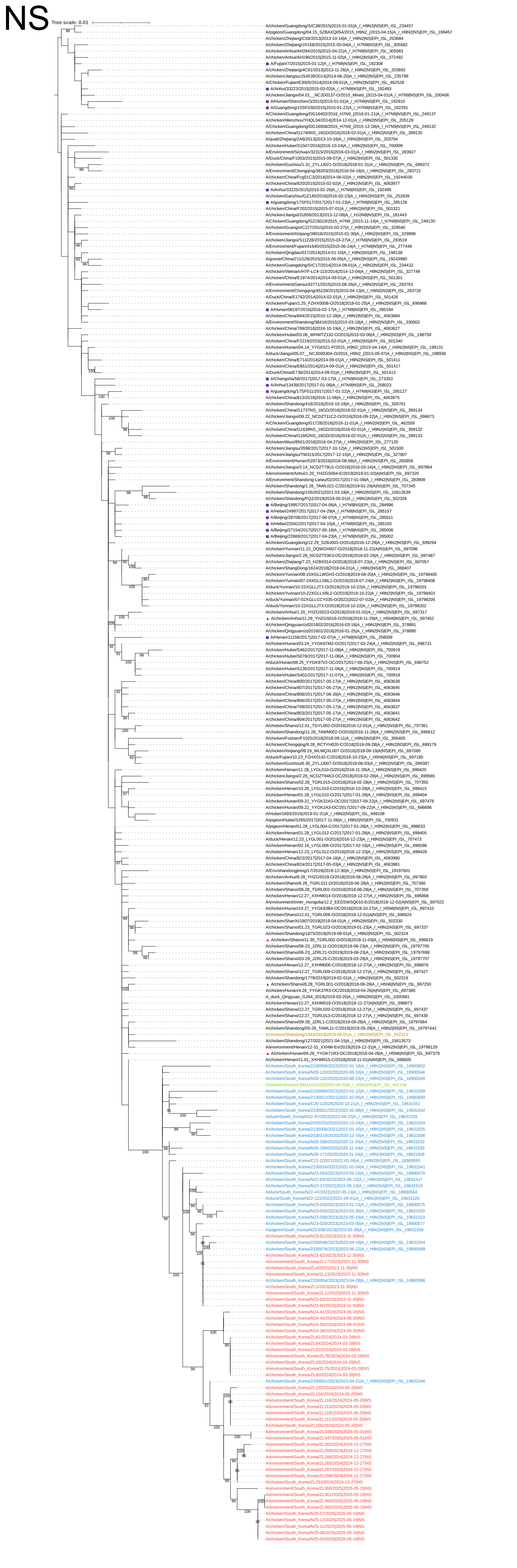
NS
Tree scale: 0.01
