# Supplementary material for: Environmental Contamination and Rapid Evolution of H9N2 Avian Influenza Virus in Live Bird Markets, South Korea, 2023–2025: Implications for Zoonotic Risk
Source: Transbound Emerg Dis. 2026 Nov 9;2026:4393527. doi: 10.1155/tbed/4393527 (PMC13748803; doi:10.1155/tbed/4393527)
Supplement: Supplementary file 3 [file TBED-2026-4393527-s004.pdf]

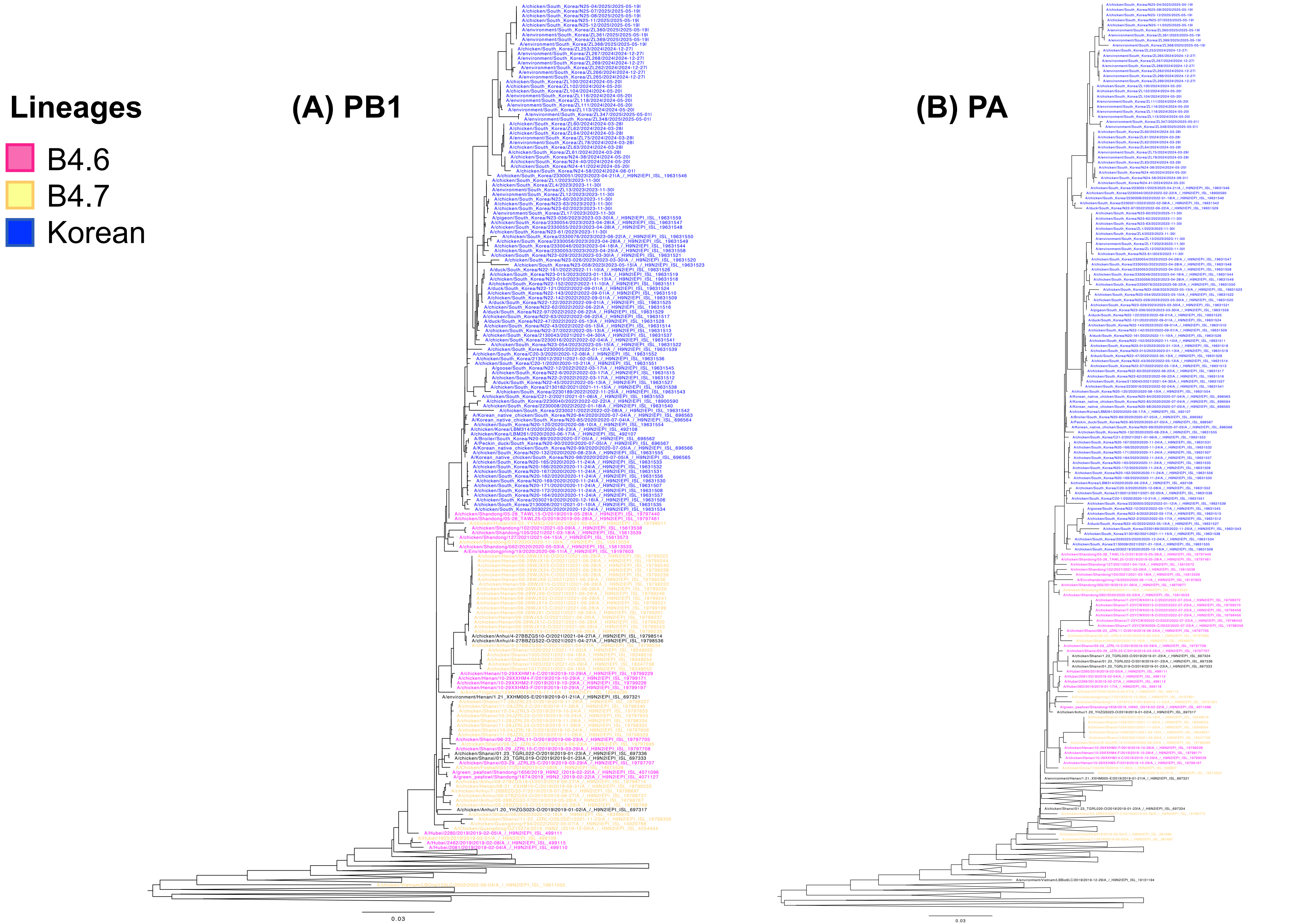

Lineages
B4.6
B4.7
Korean
(A) PB1
(B) PA
0.03
0.03
