# Supplementary material for: Environmental Contamination and Rapid Evolution of H9N2 Avian Influenza Virus in Live Bird Markets, South Korea, 2023–2025: Implications for Zoonotic Risk
Source: Transbound Emerg Dis. 2026 Nov 9;2026:4393527. doi: 10.1155/tbed/4393527 (PMC13748803; doi:10.1155/tbed/4393527)
Supplement: Supplementary file 5 [file TBED-2026-4393527-s009.pdf]

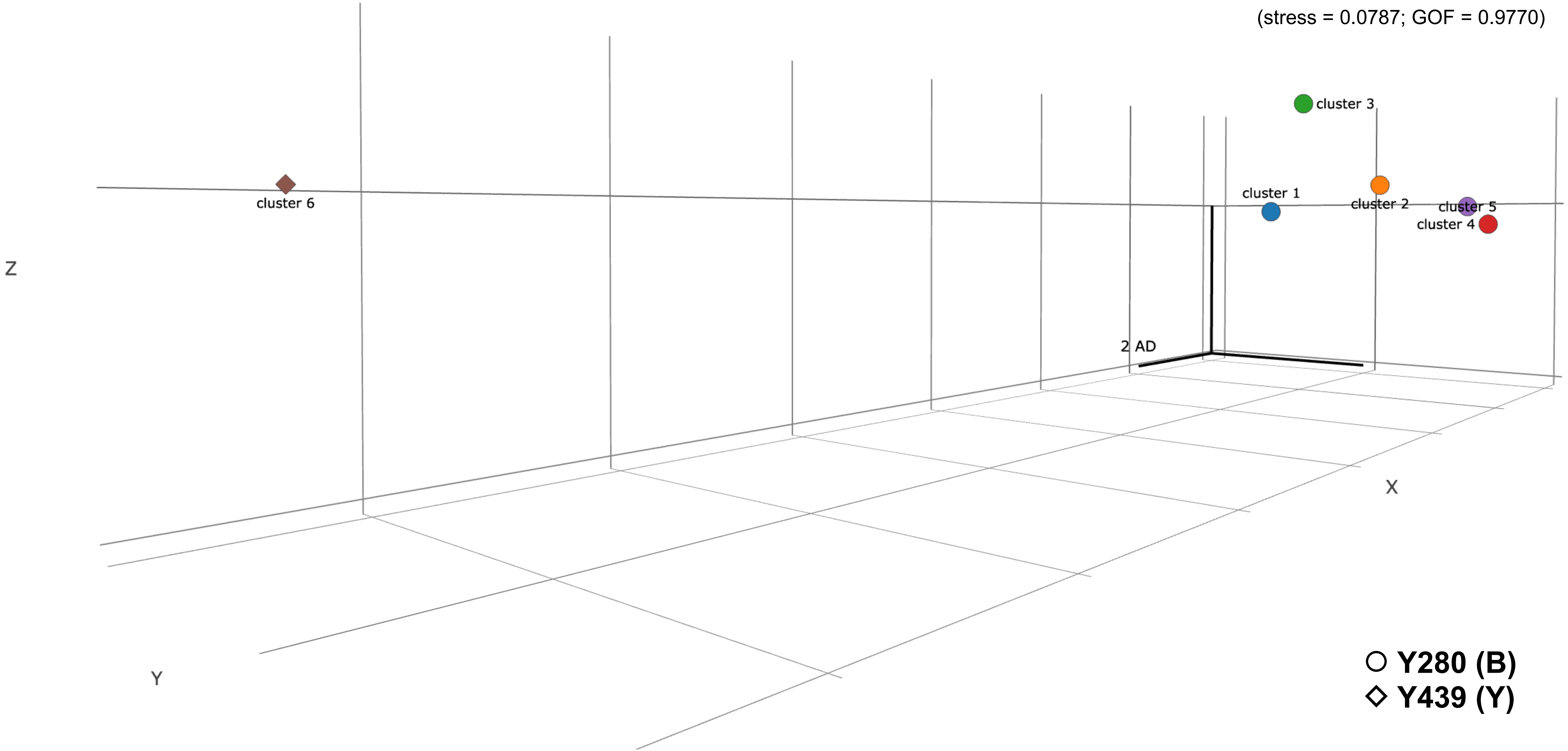
(stress = 0.0787; GOF = 0.9770)
cluster 3
cluster 6
cluster 1
cluster 2
cluster 5
cluster 4
Z
2 AD
X
Y
Y280 (B)
Y439 (Y)
