# Supplementary material for: Environmental Contamination and Rapid Evolution of H9N2 Avian Influenza Virus in Live Bird Markets, South Korea, 2023–2025: Implications for Zoonotic Risk
Source: Transbound Emerg Dis. 2026 Nov 9;2026:4393527. doi: 10.1155/tbed/4393527 (PMC13748803; doi:10.1155/tbed/4393527)
Supplement: Supplementary file 6 [file TBED-2026-4393527-s001.docx]

**Supplementary Table 4.** Pairwise sequence-based antigenic distances (ADs) among the five predicted antigenic clusters of Korean B-lineage H9N2 viruses and the Y-lineage reference strain.

|  | Cluster 1 | Cluster 2 | Cluster 3 | Cluster 4 | Cluster 5 |
| --- | --- | --- | --- | --- | --- |
| Cluster 1 | 0 |  |  |  |  |
| Cluster 2 | 1.11 | 0 |  |  |  |
| Cluster 3 | 1.11 | 1.11 | 0 |  |  |
| Cluster 4 | 2.22 | 1.11 | 2.22 | 0 |  |
| Cluster 5 | 2.22 | 1.11 | 2.22 | 1.11 | 0 |
| Y-lineage ^1^ | 11.11 | 11.11 | 11.11 | 11.11 | 11.11 |

^1^ A/chicken/Korea/01310/2001
