# Supplementary material for: Environmental Contamination and Rapid Evolution of H9N2 Avian Influenza Virus in Live Bird Markets, South Korea, 2023–2025: Implications for Zoonotic Risk
Source: Transbound Emerg Dis. 2026 Nov 9;2026:4393527. doi: 10.1155/tbed/4393527 (PMC13748803; doi:10.1155/tbed/4393527)
Supplement: Supplementary file 7 [file TBED-2026-4393527-s005.docx]

**Supplementary Table 1.** Korean B-lineage H9N2 viruses isolated and sequenced in this study.

| Isolate | Collection date | Location | Source | Sample type | Subtype | GISAID Accession ID |
| --- | --- | --- | --- | --- | --- | --- |
| N23-60 | Nov 30, 2023 | LBM A (Gyeonggi-do) | chicken | tissue | H9N2 | EPI_ISL_20134379 |
| N23-61 | Nov 30, 2023 | LBM A (Gyeonggi-do) | chicken | tissue | H9N2 | EPI_ISL_20172701 |
| N23-62 | Nov 30, 2023 | LBM A (Gyeonggi-do) | chicken | tissue | H9N2 | EPI_ISL_20172702 |
| N23-63 | Nov 30, 2023 | LBM A (Gyeonggi-do) | chicken | tissue | H9N2 | EPI_ISL_20172703 |
| N24-38 | May 20, 2024 | LBM A (Gyeonggi-do) | chicken | tissue | H9N2 | EPI_ISL_20172704 |
| N24-40 | May 20, 2024 | LBM A (Gyeonggi-do) | chicken | tissue | H9N2 | EPI_ISL_20172705 |
| N24-41 | May 20, 2024 | LBM A (Gyeonggi-do) | chicken | tissue | H9N2 | EPI_ISL_20172711 |
| N24-58 | Aug 1, 2024 | LBM A (Gyeonggi-do) | chicken | tissue | H9N2 | EPI_ISL_20172712 |
| N25-04 | May 19, 2025 | LBM A (Gyeonggi-do) | chicken | tissue | H9N2 | EPI_ISL_20172713 |
| N25-07 | May 19, 2025 | LBM A (Gyeonggi-do) | chicken | tissue | H9N2 | EPI_ISL_20172714 |
| N25-08 | May 19, 2025 | LBM A (Gyeonggi-do) | chicken | tissue | H9N2 | EPI_ISL_20172715 |
| N25-11 | May 19, 2025 | LBM A (Gyeonggi-do) | chicken | tissue | H9N2 | EPI_ISL_20172716 |
| N25-12 | May 19, 2025 | LBM A (Gyeonggi-do) | chicken | tissue | H9N2 | EPI_ISL_20172717 |
| ZL1 | Nov 30, 2023 | LBM A (Gyeonggi-do) | chicken | oropharyngeal swab | H9N2 | EPI_ISL_20243514 |
| ZL4 | Nov 30, 2023 | LBM A (Gyeonggi-do) | chicken | oropharyngeal swab | H9N2 | EPI_ISL_20243515 |
| ZL12 | Nov 30, 2023 | LBM A (Gyeonggi-do) | environment | fecal swab | H9N2 | EPI_ISL_20252431 |
| ZL13 | Nov 30, 2023 | LBM A (Gyeonggi-do) | environment | fecal swab | H9N2 | EPI_ISL_20252432 |
| ZL17 | Nov 30, 2023 | LBM A (Gyeonggi-do) | environment | drinking water | H9N2 | EPI_ISL_20252433 |
| ZL60 | Mar 28, 2024 | LBM A (Gyeonggi-do) | chicken | oropharyngeal swab | H9N2 | EPI_ISL_20252422 |
| ZL61 | Mar 28, 2024 | LBM A (Gyeonggi-do) | chicken | oropharyngeal swab | H9N2 | EPI_ISL_20252423 |
| ZL62 | Mar 28, 2024 | LBM A (Gyeonggi-do) | chicken | oropharyngeal swab | H9N2 | EPI_ISL_20252424 |
| ZL63 | Mar 28, 2024 | LBM A (Gyeonggi-do) | chicken | oropharyngeal swab | H9N2 | EPI_ISL_20252425 |
| ZL64 | Mar 28, 2024 | LBM A (Gyeonggi-do) | chicken | oropharyngeal swab | H9N2 | EPI_ISL_20252426 |
| ZL75 | Mar 28, 2024 | LBM A (Gyeonggi-do) | environment | drinking water | H9N2 | EPI_ISL_20252446 |
| ZL78 | Mar 28, 2024 | LBM A (Gyeonggi-do) | environment | drinking water | H9N2 | EPI_ISL_20252447 |
| ZL100 | May 20, 2024 | LBM A (Gyeonggi-do) | chicken | oropharyngeal swab | H9N2 | EPI_ISL_20252418 |
| ZL102 | May 20, 2024 | LBM A (Gyeonggi-do) | chicken | oropharyngeal swab | H9N2 | EPI_ISL_20252419 |
| ZL104 | May 20, 2024 | LBM A (Gyeonggi-do) | chicken | oropharyngeal swab | H9N2 | EPI_ISL_20252420 |
| ZL111 | May 20, 2024 | LBM A (Gyeonggi-do) | environment | fecal swab | H9N2 | EPI_ISL_20252427 |
| ZL113 | May 20, 2024 | LBM A (Gyeonggi-do) | environment | fecal swab | H9N2 | EPI_ISL_20252428 |
| ZL116 | May 20, 2024 | LBM A (Gyeonggi-do) | environment | drinking water | H9N2 | EPI_ISL_20252429 |
| ZL118 | May 20, 2024 | LBM A (Gyeonggi-do) | environment | drinking water | H9N2 | EPI_ISL_20252430 |
| ZL253 | Dec 27, 2024 | LBM B (Chungcheongbuk-do) | chicken | oropharyngeal swab | H9N2 | EPI_ISL_20252421 |
| ZL262 | Dec 27, 2024 | LBM B (Chungcheongbuk-do) | environment | fecal swab | H9N2 | EPI_ISL_20252434 |
| ZL265 | Dec 27, 2024 | LBM B (Chungcheongbuk-do) | environment | drinking water | H9N2 | EPI_ISL_20252435 |
| ZL266 | Dec 27, 2024 | LBM B (Chungcheongbuk-do) | environment | drinking water | H9N2 | EPI_ISL_20252436 |
| ZL267 | Dec 27, 2024 | LBM B (Chungcheongbuk-do) | environment | drinking water | H9N2 | EPI_ISL_20252437 |
| ZL268 | Dec 27, 2024 | LBM B (Chungcheongbuk-do) | environment | drinking water | H9N2 | EPI_ISL_20252438 |
| ZL269 | Dec 27, 2024 | LBM B (Chungcheongbuk-do) | environment | drinking water | H9N2 | EPI_ISL_20252439 |
| ZL347 | May 1, 2025 | LBM B (Chungcheongbuk-do) | environment | drinking water | H9N2 | EPI_ISL_20252440 |
| ZL348 | May 1, 2025 | LBM B (Chungcheongbuk-do) | environment | drinking water | H9N2 | EPI_ISL_20252441 |
| ZL360 | May 19, 2025 | LBM A (Gyeonggi-do) | environment | fecal swab | H9N2 | EPI_ISL_20252442 |
| ZL361 | May 19, 2025 | LBM A (Gyeonggi-do) | environment | fecal swab | H9N2 | EPI_ISL_20252443 |
| ZL368 | May 19, 2025 | LBM A (Gyeonggi-do) | environment | drinking water | H9N2 | EPI_ISL_20252444 |
| ZL369 | May 19, 2025 | LBM A (Gyeonggi-do) | environment | drinking water | H9N2 | EPI_ISL_20252445 |
