# Supplementary material for: Environmental Contamination and Rapid Evolution of H9N2 Avian Influenza Virus in Live Bird Markets, South Korea, 2023–2025: Implications for Zoonotic Risk
Source: Transbound Emerg Dis. 2026 Nov 9;2026:4393527. doi: 10.1155/tbed/4393527 (PMC13748803; doi:10.1155/tbed/4393527)
Supplement: Supplementary file 8 [file TBED-2026-4393527-s006.docx]

**Supplementary Table 2.** Molecular markers associated with biological properties identified in Korean B-lineage H9N2 viruses obtained in this study, listed for each isolate.

| Segment ^1^ | HA |  |  |  |  |  |  |  |  |  | PB2 |  |  |  | PB1 | PA |  |
| --- | --- | --- | --- | --- | --- | --- | --- | --- | --- | --- | --- | --- | --- | --- | --- | --- | --- |
| Substitution ^2^ | 155T  (145) ^3^ | 183N  (173) | 190T/V  (180) | 197K  (187) | 212V  (202) | 226L  (216) | 248D  (238) | 306S  (297) | 318I  (309) | 496K  (487) | 9N | 28I | 588V | 598I | 368V | 356R | 409N |
| Frequency | 45/45 | 45/45 | 6/45 | 2/45 | 45/45 | 45/45 | 45/45 | 45/45 | 2/45 | 45/45 | 1/45 | 2/45 | 45/45 | 36/45 | 45/45 | 45/45 | 45/45 |
| LBM314 ^4^ | **T** | **N** | A | R | **V** | **L** | **D** | **S** | V | **K** | D | M | **V** | V | **V** | **R** | **N** |
| N23-60 | **T** | **N** | A | R | **V** | **L** | **D** | **S** | V | **K** | D | M | **V** | V | **V** | **R** | **N** |
| N23-61 | **T** | **N** | A | R | **V** | **L** | **D** | **S** | V | **K** | D | M | **V** | V | **V** | **R** | **N** |
| N23-62 | **T** | **N** | A | R | **V** | **L** | **D** | **S** | V | **K** | D | M | **V** | V | **V** | **R** | **N** |
| N23-63 | **T** | **N** | A | R | **V** | **L** | **D** | **S** | V | **K** | D | M | **V** | V | **V** | **R** | **N** |
| N24-38 | **T** | **N** | **V** | R | **V** | **L** | **D** | **S** | V | **K** | D | M | **V** | **I** | **V** | **R** | **N** |
| N24-40 | **T** | **N** | **V** | R | **V** | **L** | **D** | **S** | V | **K** | D | M | **V** | **I** | **V** | **R** | **N** |
| N24-41 | **T** | **N** | **T** | R | **V** | **L** | **D** | **S** | V | **K** | D | M | **V** | **I** | **V** | **R** | **N** |
| N24-58 | **T** | **N** | A | R | **V** | **L** | **D** | **S** | V | **K** | D | M | **V** | **I** | **V** | **R** | **N** |
| N25-04 | **T** | **N** | A | R | **V** | **L** | **D** | **S** | V | **K** | D | M | **V** | **I** | **V** | **R** | **N** |
| N25-07 | **T** | **N** | A | R | **V** | **L** | **D** | **S** | V | **K** | D | M | **V** | **I** | **V** | **R** | **N** |
| N25-08 | **T** | **N** | A | R | **V** | **L** | **D** | **S** | V | **K** | D | M | **V** | **I** | **V** | **R** | **N** |
| N25-11 | **T** | **N** | A | R | **V** | **L** | **D** | **S** | V | **K** | D | M | **V** | **I** | **V** | **R** | **N** |
| N25-12 | **T** | **N** | A | R | **V** | **L** | **D** | **S** | V | **K** | D | M | **V** | **I** | **V** | **R** | **N** |
| ZL1 | **T** | **N** | A | R | **V** | **L** | **D** | **S** | V | **K** | D | M | **V** | V | **V** | **R** | **N** |
| ZL4 | **T** | **N** | A | R | **V** | **L** | **D** | **S** | V | **K** | D | M | **V** | V | **V** | **R** | **N** |
| ZL12 | **T** | **N** | A | R | **V** | **L** | **D** | **S** | V | **K** | D | M | **V** | V | **V** | **R** | **N** |
| ZL13 | **T** | **N** | A | R | **V** | **L** | **D** | **S** | V | **K** | D | M | **V** | V | **V** | **R** | **N** |
| ZL17 | **T** | **N** | A | R | **V** | **L** | **D** | **S** | V | **K** | D | M | **V** | V | **V** | **R** | **N** |
| ZL60 | **T** | **N** | A | R | **V** | **L** | **D** | **S** | V | **K** | D | M | **V** | **I** | **V** | **R** | **N** |
| ZL61 | **T** | **N** | A | R | **V** | **L** | **D** | **S** | V | **K** | D | M | **V** | **I** | **V** | **R** | **N** |
| ZL62 | **T** | **N** | A | R | **V** | **L** | **D** | **S** | V | **K** | D | M | **V** | **I** | **V** | **R** | **N** |
| ZL63 | **T** | **N** | A | R | **V** | **L** | **D** | **S** | V | **K** | D | M | **V** | **I** | **V** | **R** | **N** |
| ZL64 | **T** | **N** | A | R | **V** | **L** | **D** | **S** | V | **K** | D | M | **V** | **I** | **V** | **R** | **N** |
| ZL75 | **T** | **N** | A | R | **V** | **L** | **D** | **S** | V | **K** | D | M | **V** | **I** | **V** | **R** | **N** |
| ZL78 | **T** | **N** | A | R | **V** | **L** | **D** | **S** | V | **K** | D | M | **V** | **I** | **V** | **R** | **N** |
| ZL100 | **T** | **N** | A | R | **V** | **L** | **D** | **S** | V | **K** | D | M | **V** | **I** | **V** | **R** | **N** |
| ZL102 | **T** | **N** | A | R | **V** | **L** | **D** | **S** | V | **K** | D | M | **V** | **I** | **V** | **R** | **N** |
| ZL104 | **T** | **N** | A | R | **V** | **L** | **D** | **S** | V | **K** | D | M | **V** | **I** | **V** | **R** | **N** |
| ZL111 | **T** | **N** | **T** | R | **V** | **L** | **D** | **S** | V | **K** | D | M | **V** | **I** | **V** | **R** | **N** |
| ZL113 | **T** | **N** | A | R | **V** | **L** | **D** | **S** | V | **K** | D | M | **V** | **I** | **V** | **R** | **N** |
| ZL116 | **T** | **N** | A | R | **V** | **L** | **D** | **S** | V | **K** | D | M | **V** | **I** | **V** | **R** | **N** |
| ZL118 | **T** | **N** | **V** | R | **V** | **L** | **D** | **S** | V | **K** | D | M | **V** | **I** | **V** | **R** | **N** |
| ZL253 | **T** | **N** | A | R | **V** | **L** | **D** | **S** | V | **K** | D | **I** | **V** | **I** | **V** | **R** | **N** |
| ZL262 | **T** | **N** | **V** | R | **V** | **L** | **D** | **S** | V | **K** | D | M | **V** | **I** | **V** | **R** | **N** |
| ZL265 | **T** | **N** | A | R | **V** | **L** | **D** | **S** | V | **K** | D | **I** | **V** | **I** | **V** | **R** | **N** |
| ZL266 | **T** | **N** | A | R | **V** | **L** | **D** | **S** | V | **K** | D | M | **V** | **I** | **V** | **R** | **N** |
| ZL267 | **T** | **N** | A | R | **V** | **L** | **D** | **S** | V | **K** | **N** | M | **V** | **I** | **V** | **R** | **N** |
| ZL268 | **T** | **N** | A | R | **V** | **L** | **D** | **S** | V | **K** | D | M | **V** | **I** | **V** | **R** | **N** |
| ZL269 | **T** | **N** | A | R | **V** | **L** | **D** | **S** | V | **K** | D | M | **V** | **I** | **V** | **R** | **N** |
| ZL347 | **T** | **N** | A | **K** | **V** | **L** | **D** | **S** | V | **K** | D | M | **V** | **I** | **V** | **R** | **N** |
| ZL348 | **T** | **N** | A | **K** | **V** | **L** | **D** | **S** | V | **K** | D | M | **V** | **I** | **V** | **R** | **N** |
| ZL360 | **T** | **N** | A | R | **V** | **L** | **D** | **S** | V | **K** | D | M | **V** | **I** | **V** | **R** | **N** |
| ZL361 | **T** | **N** | A | R | **V** | **L** | **D** | **S** | **I** | **K** | D | M | **V** | **I** | **V** | **R** | **N** |
| ZL368 | **T** | **N** | A | R | **V** | **L** | **D** | **S** | V | **K** | D | M | **V** | **I** | **V** | **R** | **N** |
| ZL369 | **T** | **N** | A | R | **V** | **L** | **D** | **S** | **I** | **K** | D | M | **V** | **I** | **V** | **R** | **N** |

^1^ HA, hemagglutinin; PB2, polymerase basic 2 protein; PB1, polymerase basic 1 protein; PA, polymerase acidic protein.

^2^ Mutations previously linked to mammalian adaptation are indicated in bold.

^3^ Mature H3 HA numbering (mature H9 HA numbering)

^4^ A/chicken/Korea/LBM314/2020
