# Supplementary material for: Environmental Contamination and Rapid Evolution of H9N2 Avian Influenza Virus in Live Bird Markets, South Korea, 2023–2025: Implications for Zoonotic Risk
Source: Transbound Emerg Dis. 2026 Nov 9;2026:4393527. doi: 10.1155/tbed/4393527 (PMC13748803; doi:10.1155/tbed/4393527)
Supplement: Supplementary file 9 [file TBED-2026-4393527-s007.docx]

**Supplementary Table 3.** Predicted N-linked glycosylation profiles of Korean B-lineage H9N2 viruses analyzed in this study.

| Isolate | Amino acid positions ^1^ | | | | | | |
| --- | --- | --- | --- | --- | --- | --- | --- |
|  | **21 (11)** | **73 (64)** | **128 (123)** | **289 (280)** | **296 (287)** | **304 (295)** | **483 (474)** |
| LBM314 ^2^ | NSTE | NPSC | NVSY | NTTL | NVSK | NCSK | NGTY |
| N23-60 | NSTE | NPSC | NVSY | NTTL | NVSK | NCSK | NGTY |
| N23-61 | NSTE | NPSC | NVSY | NTTL | NVSK | NCSK | NGTY |
| N23-62 | NSTE | NPSC | NVSY | NTTL | NVSK | NCSK | NGTY |
| N23-63 | NSTE | NPSC | NVSY | NTTL | NVSK | NCSK | NGTY |
| N24-38 | NSTE | NPSC | NVSY | NTTL | NVSK | NCSK | NGTY |
| N24-40 | NSTE | NPSC | NVSY | NTTL | NVSK | NCSK | NGTY |
| N24-41 | NSTE | NPSC | NVSY | NTTL | NVSK | NCSK | NGTY |
| N24-58 | NSTE | NPSC | NVSY | NTTL | NVSK | NCSK | NGTY |
| N25-04 | NSTE | NPSC | NVSY | NTTL | NVSK | NCSK | NGTY |
| N25-07 | NSTE | NPSC | NVSY | NTTL | NVSK | NCSK | NGTY |
| N25-08 | NSTE | NPSC | NVSY | NTTL | NVSK | NCSK | NGTY |
| N25-11 | NSTE | NPSC | NVSY | NTTL | NVSK | NCSK | NGTY |
| N25-12 | NSTE | NPSC | NVSY | NTTL | NVSK | NCSK | NGTY |
| ZL1 | NSTE | NPSC | NVSY | NTTL | NVSK | NCSK | NGTY |
| ZL4 | NSTE | NPSC | NVSY | NTTL | NVSK | NCSK | NGTY |
| ZL12 | NSTE | NPSC | NVSY | NTTL | NVSK | NCSK | NGTY |
| ZL13 | NSTE | NPSC | NVSY | NTTL | NVSK | NCSK | NGTY |
| ZL17 | NSTE | NPSC | NVSY | NTTL | NVSK | NCSK | NGTY |
| ZL60 | NSTE | NPSC | NVSY | NTTL | NVSK | NCSK | NGTY |
| ZL61 | NSTE | NPSC | NVSY | NTTL | NVSK | NCSK | NGTY |
| ZL62 | NSTE | NPSC | NVSY | NTTL | NVSK | NCSK | NGTY |
| ZL63 | NSTE | NPSC | NVSY | NTTL | NVSK | NCSK | NGTY |
| ZL64 | NSTE | NPSC | NVSY | NTTL | NVSK | NCSK | NGTY |
| ZL75 | NSTE | NPSC | NVSY | NTTL | NVSK | NCSK | NGTY |
| ZL78 | NSTE | NPSC | NVSY | NTTL | NVSK | NCSK | NGTY |
| ZL100 | NSTE | NPSC | NVSY | NTTL | NVSK | NCSK | NGTY |
| ZL102 | NSTE | NPSC | NVSY | NTTL | NVSK | NCSK | NGTY |
| ZL104 | NSTE | NPSC | NVSY | NTTL | NVSK | NCSK | NGTY |
| ZL111 | NSTE | NPSC | NVSY | NTTL | NVSK | NCSK | NGTY |
| ZL113 | NSTE | NPSC | NVSY | NTTL | NVSK | NCSK | NGTY |
| ZL116 | NSTE | NPSC | NVSY | NTTL | NVSK | NCSK | NGTY |
| ZL118 | NSTE | NPSC | NVSY | NTTL | NVSK | NCSK | NGTY |
| ZL253 | NSTE | NPSC | NVSY | NTTL | NVSK | NCSK | NGTY |
| ZL262 | NSTE | NPSC | NVSY | NTTL | NVSK | NCSK | NGTY |
| ZL265 | NSTE | NPSC | NVSY | NTTL | NVSK | NCSK | NGTY |
| ZL266 | NSTE | NPSC | NVSY | NTTL | NVSK | NCSK | NGTY |
| ZL267 | NSTE | NPSC | NVSY | NTTL | NVSK | NCSK | NGTY |
| ZL268 | NSTE | NPSC | NVSY | NTTL | NVSK | NCSK | NGTY |
| ZL269 | NSTE | NPSC | NVSY | NTTL | NVSK | NCSK | NGTY |
| ZL347 | NSTE | NPSC | NVSY | NTTL | NVSK | NCSK | NGTY |
| ZL348 | NSTE | NPSC | NVSY | NTTL | NVSK | NCSK | NGTY |
| ZL360 | NSTE | NPSC | NVSY | NTTL | NVSK | NCSK | NGTY |
| ZL361 | NSTE | NPSC | NVSY | NTTL | NVSK | NCSK | NGTY |
| ZL368 | NSTE | NPSC | NVSY | NTTL | NVSK | NCSK | NGTY |
| ZL369 | NSTE | NPSC | NVSY | NTTL | NVSK | NCSK | NGTY |

^1^ Mature H3 HA numbering (mature H9 HA numbering)

^2^ A/chicken/Korea/LBM314/2020
